# Postnatal Expression Profile of microRNAs Associated with Cardiovascular and Cerebrovascular Diseases in Children at the Age of 3 to 11 Years in Relation to Previous Occurrence of Pregnancy-Related Complications

**DOI:** 10.3390/ijms20030654

**Published:** 2019-02-02

**Authors:** Ilona Hromadnikova, Katerina Kotlabova, Lenka Dvorakova, Ladislav Krofta, Jan Sirc

**Affiliations:** 1 Department of Molecular Biology and Cell Pathology, Third Faculty of Medicine, Charles University, 10000 Prague, Czech Republic; katerina.kotlabova@lf3.cuni.cz (K.K.); lenka.dvorakova@lf3.cuni.cz (L.D.); 2 Institute for the Care of the Mother and Child, Third Faculty of Medicine, Charles University, 14700 Prague, Czech Republic; ladislav.krofta@upmd.eu (L.K.); jan.sirc@upmd.eu (J.S.)

**Keywords:** Body mass index (BMI), cardiovascular/cerebrovascular diseases, cardiovascular risk, children, echocardiography, microRNA expression, pregnancy complications, prehypertension/hypertension, primary prevention, screening

## Abstract

Children descending from pregnancies complicated by gestational hypertension (GH), preeclampsia (PE) or fetal growth restriction (FGR) have a lifelong cardiovascular risk. The aim of the study was to verify if pregnancy complications induce postnatal alterations in gene expression of microRNAs associated with cardiovascular/cerebrovascular diseases. Twenty-nine microRNAs were assessed in peripheral blood, compared between groups, and analyzed in relation to both aspects, the current presence of cardiovascular risk factors and cardiovascular complications and the previous occurrence of pregnancy complications with regard to the clinical signs, dates of delivery, and Doppler ultrasound examination. The expression profile of miR-21-5p differed between controls and children with a history of uncomplicated pregnancies with abnormal clinical findings. Abnormal expression profile of multiple microRNAs was found in children affected with GH (miR-1-3p, miR-17-5p, miR-20a-5p, miR-21-5p, miR-23a-3p, miR-26a-5p, miR-29a-3p, miR-103a-3p, miR-125b-5p, miR-126-3p, miR-133a-3p, miR-146a-5p, miR-181a-5p, miR-195-5p, and miR-342-3p), PE (miR-1-3p, miR-20a-5p, miR-20b-5p, miR-103a-3p, miR-133a-3p, miR-342-3p), and FGR (miR-17-5p, miR-126-3p, miR-133a-3p). The index of pulsatility in the ductus venosus showed a strong positive correlation with miR-210-3p gene expression in children exposed to PE and/or FGR. Any of changes in epigenome (up-regulation of miR-1-3p and miR-133a-3p) that were induced by pregnancy complications are long-acting and may predispose children affected with GH, PE, or FGR to later development of cardiovascular/cerebrovascular diseases. Novel epigenetic changes (aberrant expression profile of microRNAs) appeared in a proportion of children that were exposed to GH, PE, or FGR. Screening of particular microRNAs may stratify a highly risky group of children that might benefit from implementation of early primary prevention strategies.

## 1. Introduction

There exists a progressive increase of data linking specific levels of risk factors in prenatal life with cardiovascular disease outcomes in children and adolescents. Recent epidemiologic and experimental data substantially indicate that children descending from pregnancies complicated by gestational hypertension (GH), preeclampsia (PE), or fetal growth restriction (FGR) have an unique, lifetime cardiovascular risk profile that is present from early life. Young offspring of pregnancies complicated by GH or PE already have increased body mass index (BMI) and wider waist circumference [1,2]. Systolic and/or diastolic blood pressures (BP) were also reported to be higher in offspring of mothers with GH or PE compared with offspring of mothers without hypertensive disorders of pregnancy [1,2,3,4,5]. These differences were even consistent till the age of 18 years, as the patterns of blood pressure change did not differ between children of hypertensive and normotensive pregnancies [6]. Offspring from pregnancies with early PE (onset < 34 weeks) had at the age of 6 to 13 years a higher systolic BP and higher nocturnal systolic and diastolic BP than those born to late onset PE [7]. Maternal central pulsatile BP components (systolic BP and pulse pressure) during pregnancy were associated with higher BP in the offspring of women with PE. This positive correlation was already evident at 3 years old children [8]. These results suggested a possible association between maternal hypertensive disorders of pregnancy and offspring BP that may be driven by genetics or familial non-genetic risk factors particular to BP [5]. In addition, PE leaves a persistent alteration in the systemic and the pulmonary circulation of the children. Pulmonary artery pressure was roughly 30% higher and flow-mediated dilation was 30% smaller in children of PE-affected mothers than in controls. This alteration predisposes children to exaggerated hypoxic pulmonary hypertension already during childhood and may cause premature cardiovascular disease in the systemic circulation at some time in the future [9].

Sarvari et al. [10] reported that FGR induced cardiac remodeling persists until preadolescence (8–12 years of age) with findings similar to those reported in their prenatal life and childhood. Echocardiography and three-dimensional shape computational analysis revealed a more spherical and smaller hearts, decreased longitudinal motion and impaired relaxation in children affected with FGR [10]. Yiallourou et al. [11] revealed that preterm FGR children aged 5–12 years had smaller left ventricular lengths, ascending aorta, and left ventricular outflow tract diameter and vascular compliance was positively correlated with gestational age. These findings result in the hypothesis of FGR caused primary cardiac programming for explaining the association between low birth weight and later cardiovascular risk [10].

Birth weight influences childhood BP as well, but the effects may vary depending on ethnic group. FGR and early gestational age were associated with higher BP in white but not African American children at the age of 5 years [12]. 

Altogether, childhood obesity, hypertension, diabetes, and cardiac remodeling are the most prevalent intermediate and long-standing health consequences of undernutrition of the fetus caused by insufficiency of the placenta [13,14]. 

Although, health factors such as lipid levels, BMI, and BP normally change with age, growth, and development, children affected by pregnancy complications including GH, PE, and FGR represent a population that would benefit from implementation of early primary prevention strategies. At least modification of diet and physical activity are first-line interventions. Appropriate pharmacological intervention may be also considered when lifestyle change is not successful [15]. 

The goal of the study was to evaluate an epigenetic profile for the detection of cardiovascular risk in whole peripheral blood of children at the age of 3 to 11 years born out of pregnancies complicated by GH, PE, and FGR. Postnatal epigenetic profiling of microRNAs playing a role in pathogenesis of diverse cardiovascular/cerebrovascular diseases (miR-1-3p, miR-16-5p, miR-17-5p, miR-20a-5p, miR-20b-5p, miR-21-5p, miR-23a-3p, miR-24-3p, miR-26a-5p, miR-29a-3p, miR-92a-3p, miR-100-5p, miR-103a-3p, miR-125b-5p, miR-126-3p, miR-130b-3p, miR-133a-3p, miR-143-3p, miR-145-5p, miR-146a-5p, miR-155-5p, miR-181a-5p, miR-195-5p, miR-199a-5p, miR-210-3p, miR-221-3p, miR-342-3p, miR-499a-5p, and miR-574-3p) was the subject of our interest. We focused mainly on those microRNAs known to be involved in the onset of dyslipidaemia [16,17], hypertension [18,19,20], vascular inflammation [21,22], insulin resistance and diabetes [23], atherosclerosis [24,25], angiogenesis [26,27], coronary artery disease [19,22,26,28], myocardial infarction and heart failure [18,29,30,31,32], stroke [33], intracranial aneurysm [34], pulmonary arterial hypertension [35], and peripartum cardiomyopathy [36].

MicroRNAs are the members of the family of small noncoding RNAs that regulate expression of genes at the posttranscriptional level by blocking translation or degrading of target messenger RNA [37]. MicroRNA analyses indicate that under pathological conditions a variety of tissues display diverse microRNA expression profiles, which may be used in clinical diagnostics [38]. Recent studies have demonstrated that GH, PE, and FGR are associated with alterations in microRNA expression in the maternal circulation, placenta, and umbilical cord blood [39,40,41,42,43]. 

To the best of our knowledge, any study on expression profiling of microRNAs associated with cardiovascular and cerebrovascular diseases in whole peripheral blood of children descending from GH, PE, and FGR affected pregnancies has not been carried out. 

## 2. Results

### 2.1. Distribution of Children Descending from Normal Pregnancies into Groups Based on Clinical Examination and Consequent Findings

Children descending from normal pregnancies were divided into two groups based on the results of examination and clinical findings. Children already dispesarized in the department of pediatric cardiology (*n* = 8) and those ones indicated by the sonographer to have valve problems and heart defects tricuspid valve regurgitation (*n* = 8), mitral valve regurgitation (*n* = 1), pulmonary valve regurgitation (*n* = 2), bicuspid aortic valve regurgitation (*n* = 1), ventricular septum defect (*n* = 1), atrial septum defect (*n* = 1), foramen ovale apertum (*n* = 5), arrhythmia (*n* = 1) constituted particular group together with children confirmed over several visits to have a high BP (*n* = 16) (systolic blood pressure (SBP) and/or diastolic blood pressure (DBP) ≥ 90th percentile evaluated by the Age-Based Pediatric Blood Pressure Reference Charts calculator) and/or high BMI (*n* = 9) (BMI 85th percentile evaluated by the BMI Percentile Calculator for Child and Teens). Overall, this group consisted of 38 children (43.18%). The second group consisted from 50 children with normal anamnesis, normal BP, normal BMI, and normal reference values of echocardiographic measurements.

### 2.2. Up-Regulation of miR-21-5p in Children Descending from Normal Pregnancies that are Overweight/Obese, Prehypertensive/Hypertensive and/or have Abnormal Echocardiogram Findings

**Figure 1:**
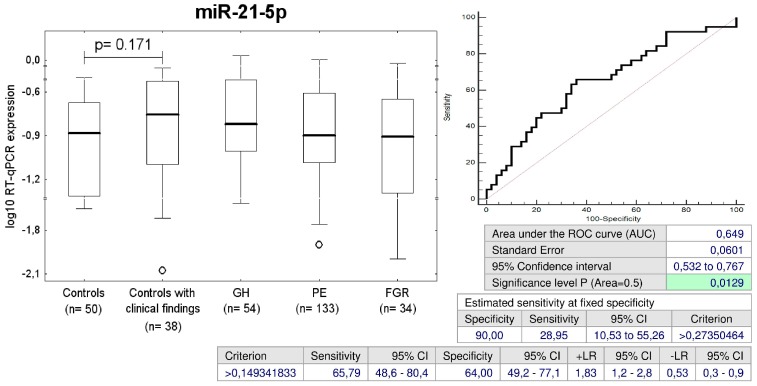
Up-regulation of miR-21-5p in children descending from normal pregnancies that are overweight/obese, prehypertensive/hypertensive and/or have abnormal echocardiogram findings. GH: gestational hypertension; PE: preeclampsia; FGR: fetal growth restriction; ROC: receivers operating characteristic; AUC: Area under the curve; +LR: likelihood ratio positive; –LR: likelihood ratio negative.

Since we identified within the group of children descending from normal pregnancies those ones with cardiac findings who were already dispesarized in the department of pediatric cardiology, or those ones who were overweight/obese, had prehypertension/hypertension, and/or abnormal echocardiogram findings, we compared the microRNA profile of this group to that one consisting of children with normal anamnesis, normal BP, normal BMI and normal reference values of echocardiographic measurements. The performance of receivers operating characteristic (ROC) curve analysis revealed that only miR-21-5p differentiated children descending from normal pregnancies in dependence on the presence or absence of postnatal abnormal clinical findings with a sensitivity of 28.95% at a specificity of 90.0% (Figure 1). 

### 2.3. Dysregulation of Cardiovascular/Cerebrovascular Disease Associated microRNAs in Children Descending from Complicated Pregnancies 

MicroRNA gene expression was compared between children descending from normal and complicated pregnancies (GH, PE, and FGR). MicroRNA gene expression was analyzed in relation to both aspects, the current presence of cardiovascular risk factors (overweight/obesity and/or prehypertension/hypertension) and cardiovascular complications (valve problems and heart defects) and the previous occurrence of pregnancy-related complications with respect to clinical signs (mild versus severe preeclampsia), dates of delivery (< and > 34 weeks in case of PE, < and > 32 weeks in case of FGR, respectively). The association between microRNA gene expression and Doppler ultrasonography parameters (pulsatility index (PI) in the umbilical artery, PI in the middle cerebral artery, the cerebroplacental ratio, PI in the uterine artery, PI in the ductus venosus, and the presence of unilateral or bilateral diastolic notch in the uterine artery) was analyzed in the group of complicated pregnancies (PE and/or FGR). Just the results that reached a statistical significance or displayed a trend toward aberrant microRNA expression profile in complicated cases are presented below.

### 2.4. Multiple microRNAs are Up-Regulated in Children Descending from GH Pregnancies 

**Figure 2:**
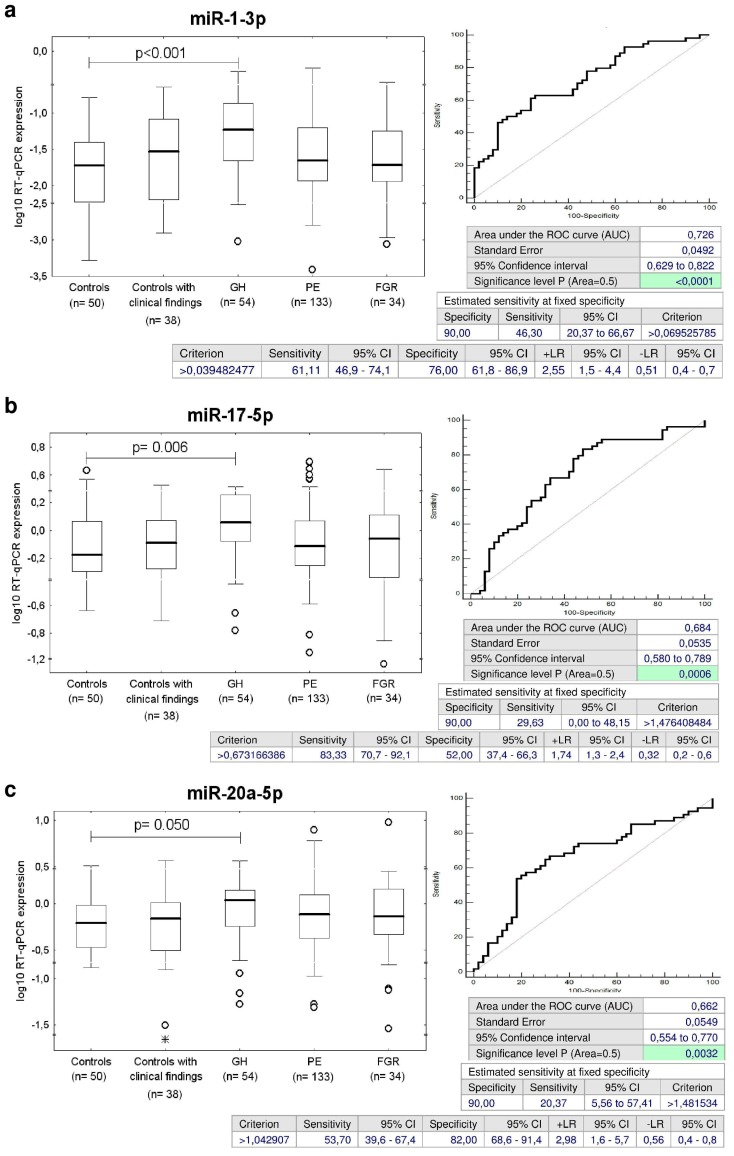

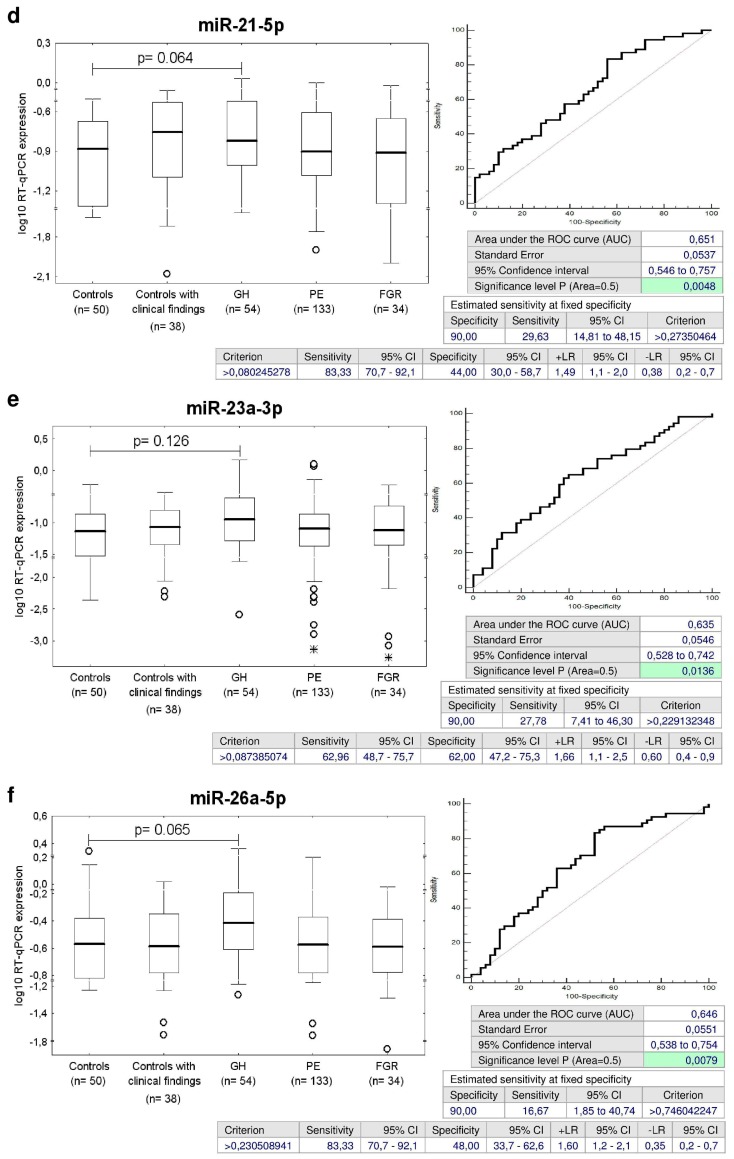

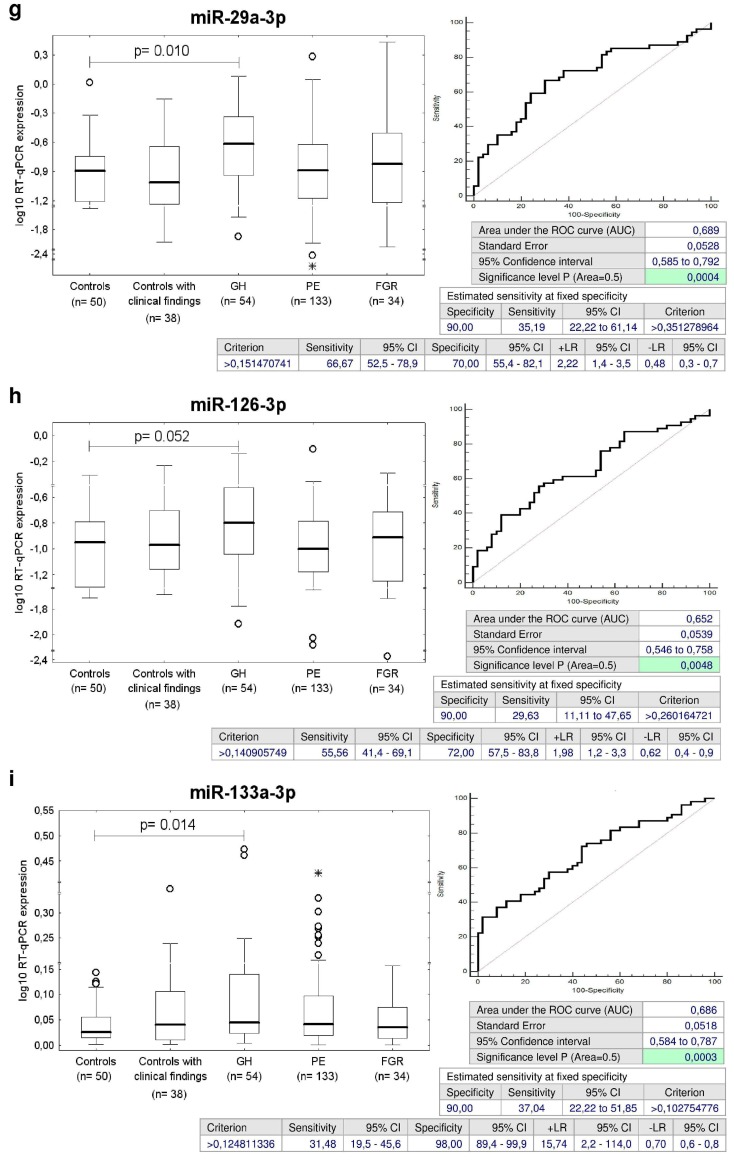

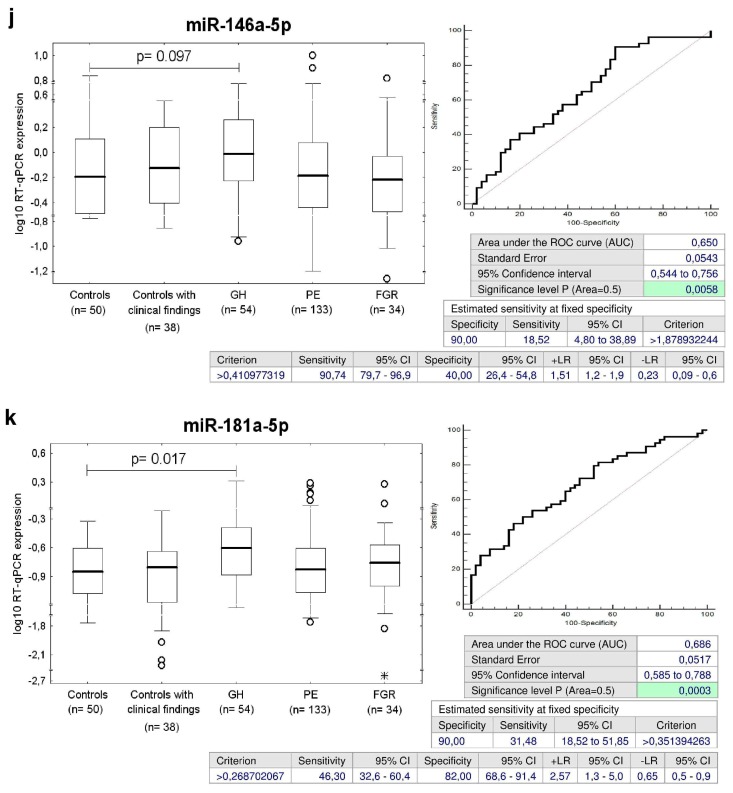
Postnatal microRNA expression profile in children descending from GH pregnancies. (**a**–**k**) Up-regulation of miR-1-3p, miR-17-5p, miR-20a-5p, miR-21-5p, miR-23a-3p, miR-26a-5p, miR-29a-3p, miR-126-3p, miR-133a-3p, miR-146a-5p, and miR-181a-5p was observed in children descending from GH pregnancies when the comparison to the controls was performed.

The ROC curve analysis revealed a significant up-regulation of miR-1-3p, miR-17-5p, miR-20a-5p, miR-21-5p, miR-23a-3p, miR-26a-5p, miR-29a-3p, miR-126-3p, miR-133a-3p, miR-146a-5p, and miR-181a-5p for children descending from GH pregnancies when the comparison to the controls was performed (Figure 2). 

The sensitivity at 10.0% false positive rate (FPR) for miR-1-3p (46.3%), miR-17-5p (29.63%), miR-20a-5p (20.37%), miR-21-5p (29.63%), miR-23a-3p (27.78%), miR-26a-5p (16.67%), miR-29a-3p (35.19%), miR-126-3p (29.63%), miR-133a-3p (37.04%), miR-146a-5p (18.52%), and miR-181a-5p (31.48%) was found (Figure 2).

### 2.5. Up-Regulation of miR-21-5p, miR-23a-3p, miR-26a-5p, miR-103a-3p, miR-125b-5p, miR-195-5p, and miR-342-3p in Children with Normal Postnatal Clinical Findings Descending from GH Pregnancies 

**Figure 3:**
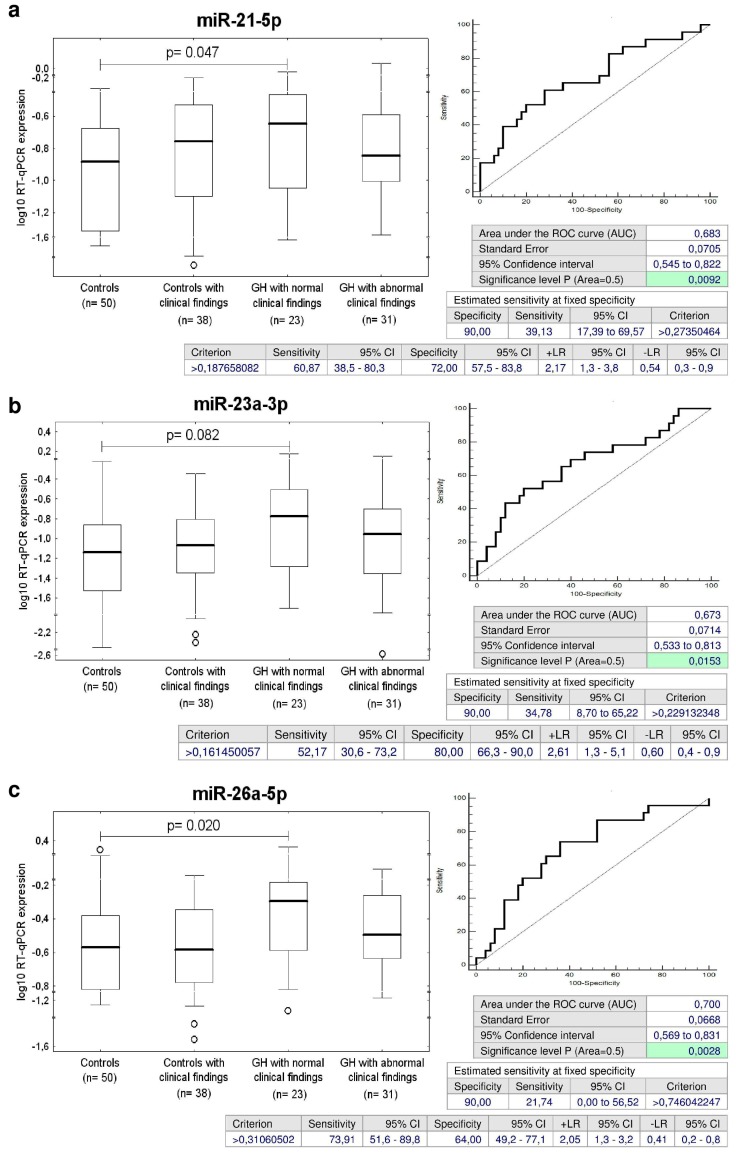

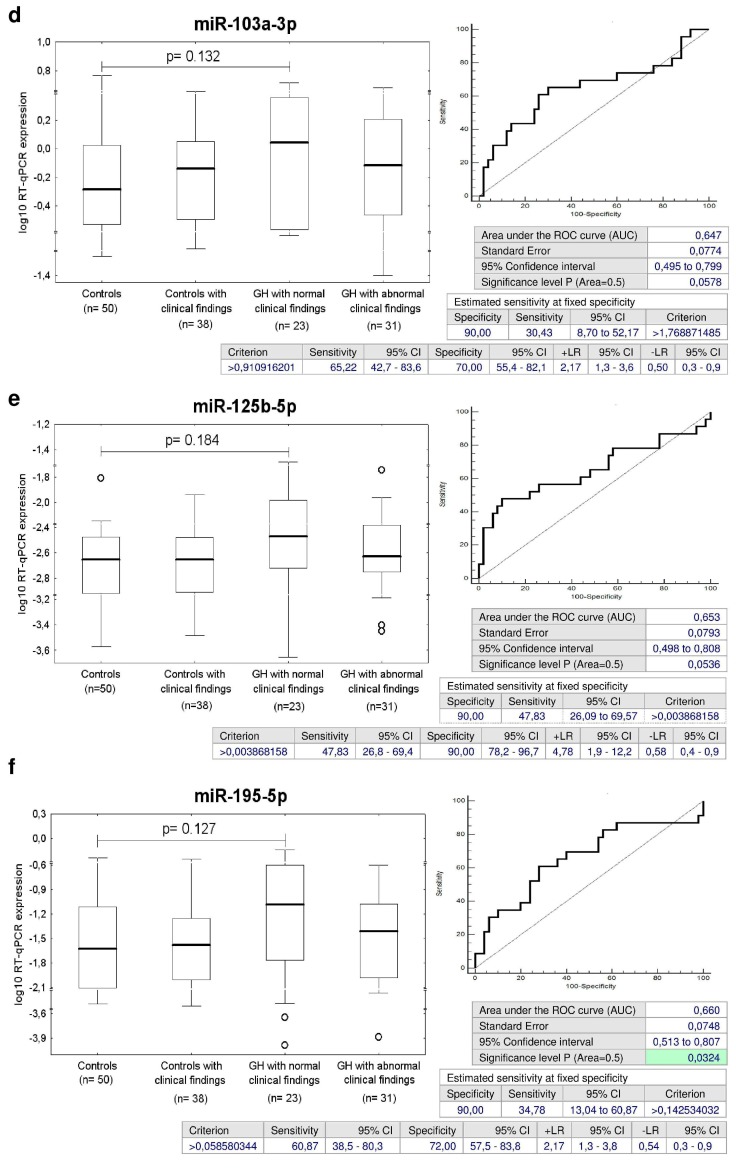

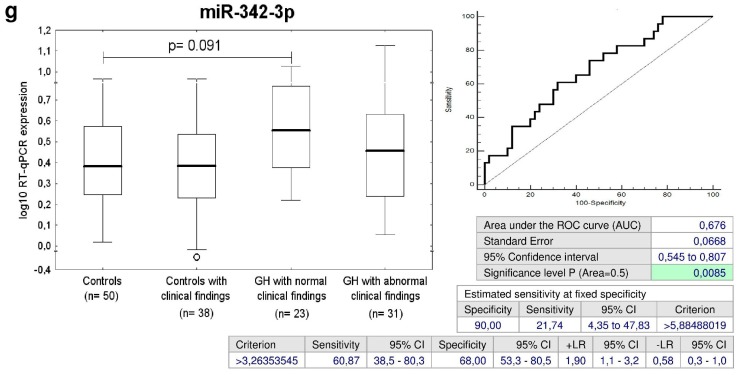
Postnatal microRNA expression profile in children with normal postnatal clinical findings descending from GH pregnancies. (**a**–**g**) Up-regulation of miR-21-5p, miR-23a-3p, miR-26a-5p, miR-103a-3p, miR-125b-5p, miR-195-5p and miR-342-3p was observed in children with normal postnatal clinical findings descending from GH pregnancies.

Concurrently, it was observed that the expression of miR-21-5p, miR-23a-3p, miR-26a-5p, miR-103a-3p, miR-125b-5p, miR-195-5p, and miR-342-3p differed significantly between the groups of children affected with GH with normal postnatal clinical findings and the controls (Figure 3). The sensitivity of individual microRNAs at 10.0% FPR was the following: miR-21-5p (39.13%), miR-23a-3p (34.78%), miR-26a-5p (21.74%), miR-103a-3p (30.43%), miR-125b-5p (47.83%), miR-195-5p (34.78%), and miR-342-3p (21.74%) (Figure 3). 

**Figure 4:**
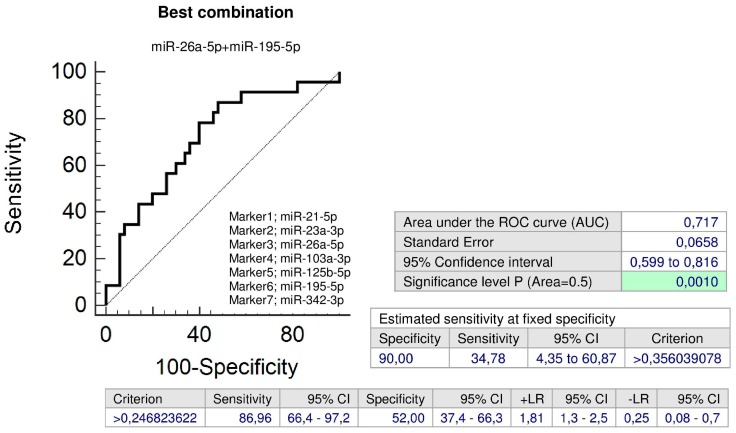
Combined postnatal screening of microRNAs in the identification of children with normal postnatal clinical findings descending from GH pregnancies. Postnatal combined screening of miR-26a-5p and miR-195-5p showed the highest accuracy for the identification of children with normal clinical findings with a prior exposure to GH at a higher risk of later development of cardiovascular/cerebrovascular diseases.

Screening based on the combination of miR-26a-5p and miR-195-5p showed the highest accuracy for children with normal clinical findings with a prior exposure to GH (AUC 0.717, *p* = 0.001, sensitivity 86.96%, specificity 52.0%, cut off > 0.246824). It was able to identify 34.78% children with an increased cardiovascular risk at 10.0% FPR (Figure 4).

### 2.6. Up-Regulation of miR-20a-5p in Children with Abnormal Postnatal Clinical Findings Descending from GH Pregnancies

**Figure 5:**
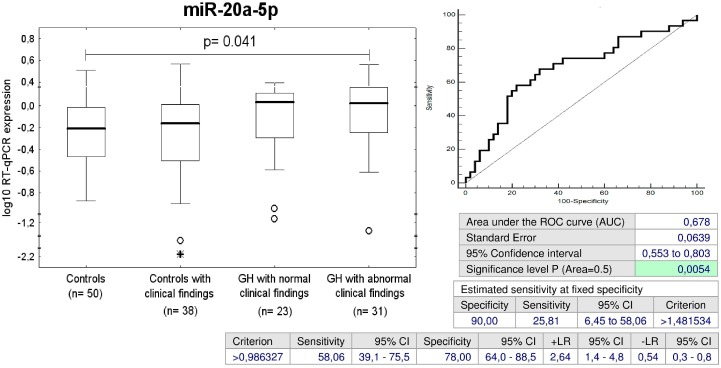
Increased expression of miR-20a-5p in children with abnormal postnatal clinical findings descending from GH pregnancies. Increased expression of miR-20a-5p was found in children with abnormal postnatal clinical findings descending from GH pregnancies.

Overall, the group with abnormal postnatal clinical findings consisted of 31/54 children (57.41%) exposed to GH, 5 children already dispesarized in the department of pediatric cardiology, 12 children with abnormal echocardiographic findings (6 tricuspid valve regurgitation, 1 mitral valve regurgitation, 3 pulmonary valve regurgitation, 1 bicuspid aortic valve regurgitation, 1 ventricular septum defect, 2 foramen ovale apertum), 17 children with prehypertension/hypertension, and 3 children with high BMI. miR-20a-5p expression differed between the groups of children affected with GH with abnormal postnatal clinical findings and the controls. miR-20a-5p differentiated between the children descending from pregnancies affected with GH with abnormal postnatal clinical findings and the controls with a sensitivity of 25.81% at 10.0% FPR (Figure 5).

### 2.7. Up-Regulation of miR-1-3p, miR-17-5p, miR-29a-3p, miR-126-3p, miR-133a-3p, miR-146a-5p, and miR-181a-5p in Children Descending from GH Pregnancies Irrespective of Postnatal Clinical Findings

**Figure 6:**
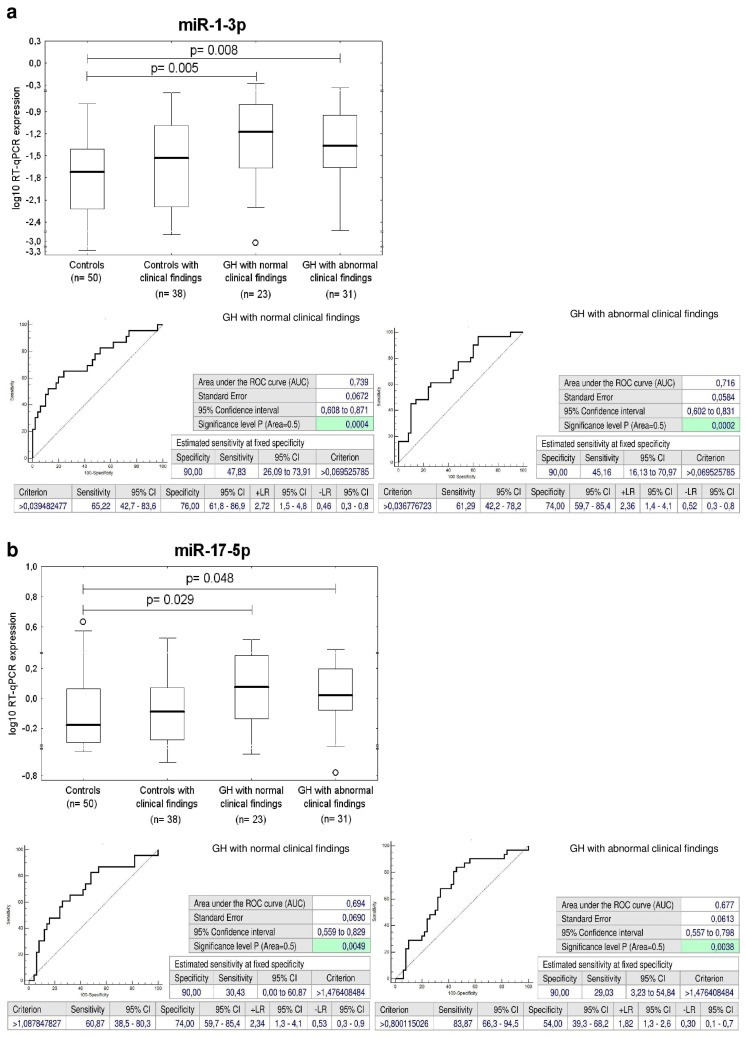

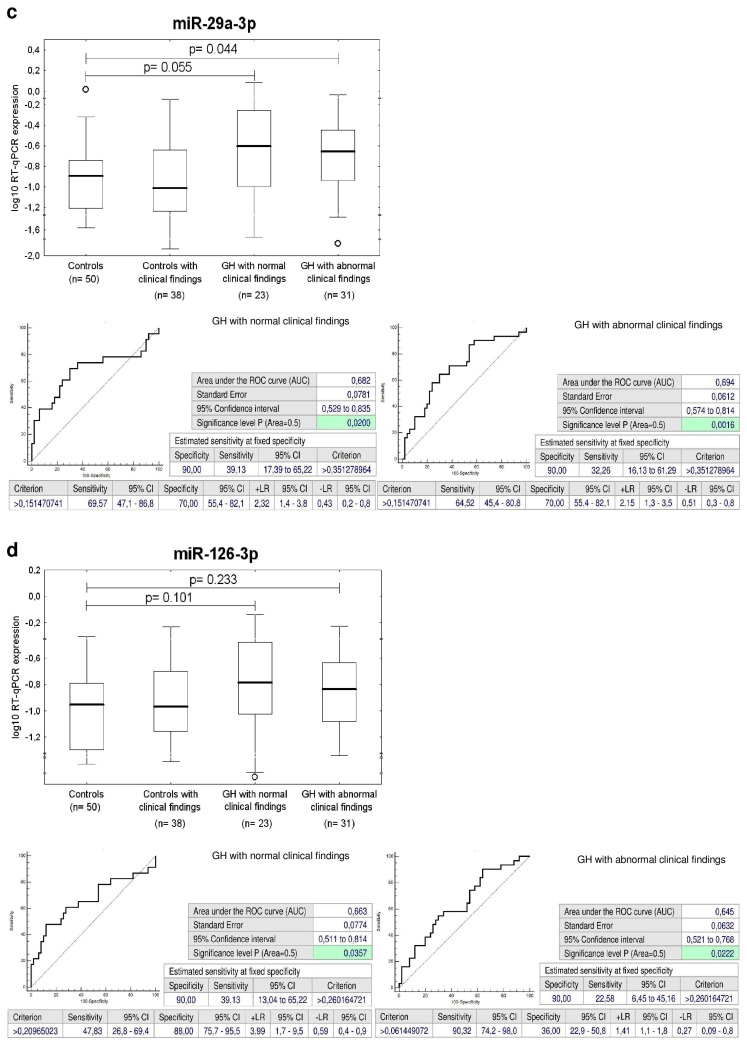

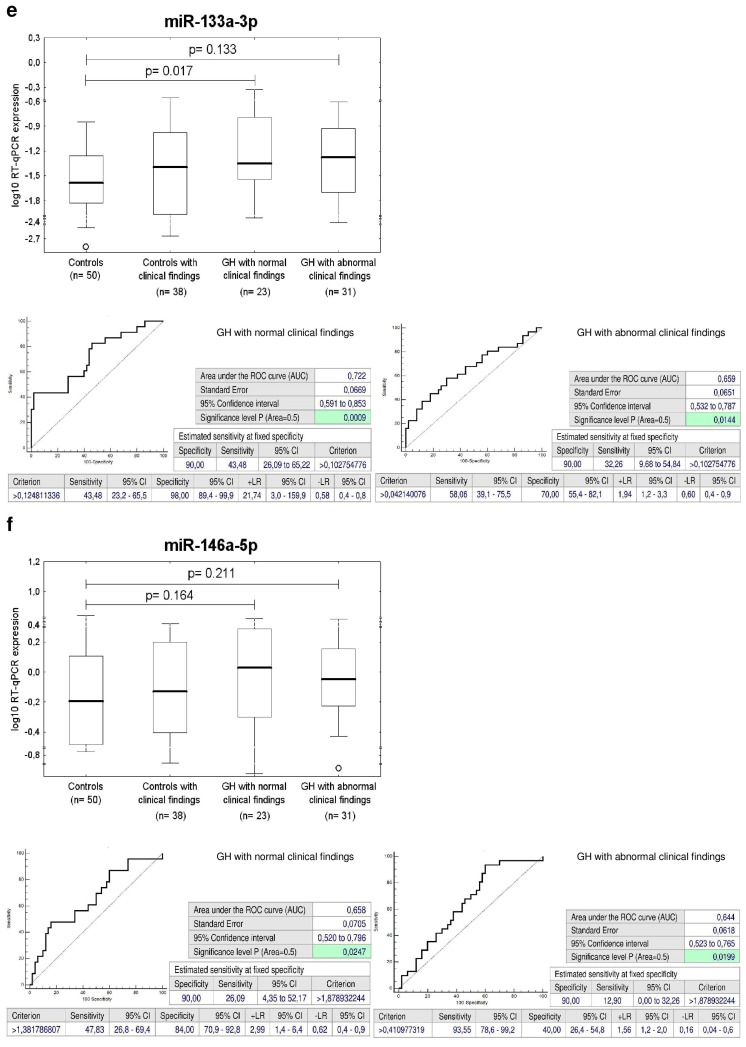

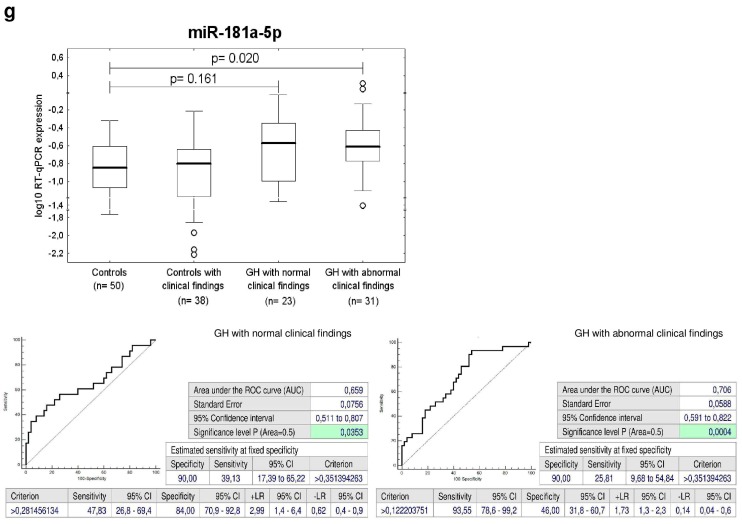
Postnatal microRNA expression profile in children descending from GH pregnancies irrespective of postnatal clinical findings. (**a**–**g**) Increased expression of miR-1-3p, miR-17-5p, miR-29a-3p, miR-126-3p, miR-133a-3p, miR-146a-5p, and miR-181a-5p was observed in children descending from GH pregnancies with normal or abnormal postnatal clinical findings. The ROC curve analysis showed the difference in microRNA gene expression between the controls and the group of children exposed to GH with postnatal normal clinical findings or children with a prior exposure to GH that already developed any cardiovascular complication (valve problems and heart defects) or were identified to be overweight/obese and/or prehypertensive/hypertensive.

The ROC curve analysis showed the difference in gene expression of miR-1-3p, miR-17-5p, miR-29a-3p, miR-126-3p, miR-133a-3p, miR-146a-5p, and miR-181a-5p between the controls and the group of children exposed to GH with postnatal normal clinical findings or children with a prior exposure to GH that already developed any cardiovascular complication (valve problems and heart defects) or were identified to be overweight/obese and/or prehypertensive/hypertensive (Figure 6). The sensitivity at 10.0% FPR for miR-1-3p (47.83% versus 45.16%) and miR-17-5p (30.43% versus 29.03%) for children descending from GH pregnancies with postnatal normal and abnormal clinical findings was approximately equal (Figure 6). The sensitivity at 10.0% FPR for miR-29a-3p (39.13% versus 32.26%), miR-126-3p (39.13% versus 22.58%), miR-133a-3p (43.48% versus 32.26%), miR-146a-5p (26.09% versus 12.9%), and miR-181a-5p (39.13% versus 25.81%) was slightly higher for children descending from GH pregnancies with postnatal normal clinical findings when compared to those ones with postnatal abnormal clinical findings (Figure 6). 

**Figure 7:**
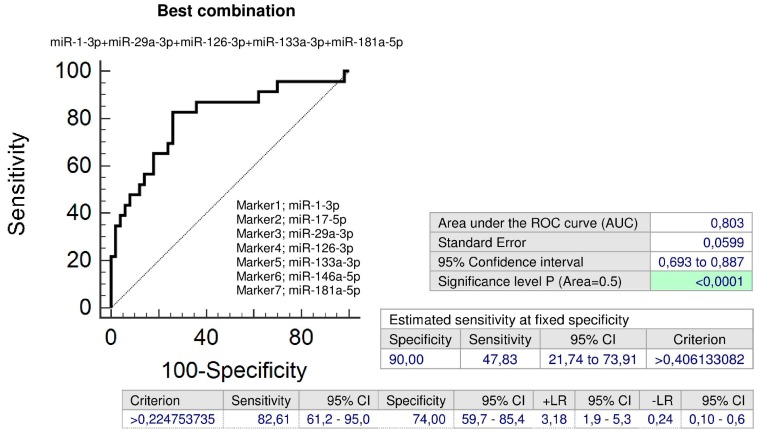
Combined postnatal screening of microRNAs in the identification of children with normal postnatal clinical findings descending from GH pregnancies. Postnatal combined screening of miR-1-3p, miR-29a-3p, miR-126-3p, miR-133a-3p and miR-181a-5p showed the highest accuracy for the identification of children with normal clinical findings with a prior exposure to GH at a higher risk of later development of cardiovascular/cerebrovascular diseases.

Combined screening of miR-1-3p, miR-29a-3p, miR-126-3p, miR-133a-3p, and miR-181a-5p showed the highest accuracy for children with a prior exposure to GH with normal clinical findings (AUC 0.803, *p* < 0.001, sensitivity 82.61%, specificity 74.0%, cut off > 0.224754). It was able to identify 47.83% children with an increased cardiovascular risk at 10.0% FPR (Figure 7). 

**Figure 8:**
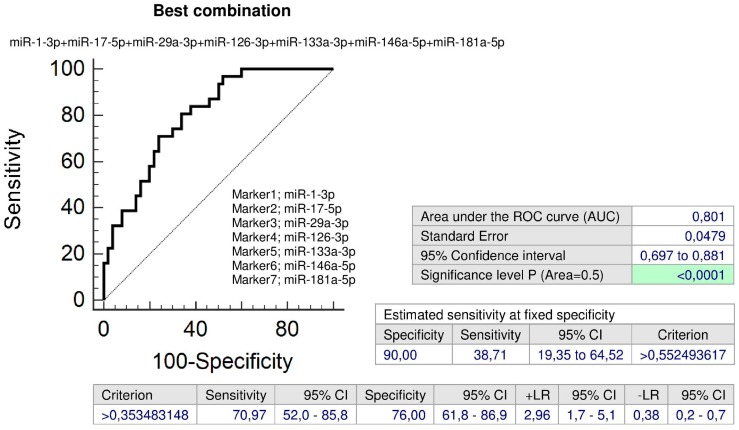
Combined postnatal screening of microRNAs in the identification of children with abnormal postnatal clinical findings descending from GH pregnancies. Postnatal combined screening of miR-1-3p, miR-17-5p, miR-29a-3p, miR-126-3p, mir-133a-3p, miR-146a-5p, and miR-181a-5p showed the highest accuracy for the identification of children with abnormal clinical findings with a prior exposure to GH at an increased risk of later onset of cardiovascular/cerebrovascular diseases.

In addition, the combination of all seven microRNA biomarkers (miR-1-3p, miR-17-5p, miR-29a-3p, miR-126-3p, miR-133a-3p, miR-146a-5p, and miR-181a-5p) may be used to identify children with a prior exposure to GH with abnormal clinical findings (AUC 0.801, *p* < 0.001, sensitivity 70.97%, specificity 76.0%, cut off > 0.353483). It was able to identify 38.71% children with an increased cardiovascular risk at 10.0% FPR (Figure 8).

### 2.8. Cardiovascular/Cerebrovascular Disease Associated microRNAs are Dysregulated in Children Descending from PE Pregnancies 

Overall, the group with abnormal postnatal clinical findings consisted of 63/133 children (47.37%) exposed to PE 19 children already dispesarized in the department of pediatric cardiology, 18 children with abnormal echocardiographic findings (7 tricuspid valve regurgitation, 2 mitral valve regurgitation, 1 pulmonary valve regurgitation, 1 ventricular septum defect, 1 atrial septum defect, 1 hypertrophic ventricular septum, 9 foramen ovale apertum, and 1 arrhythmia), 36 children with prehypertension/hypertension and 5 children with high BMI. Abnormal clinical findings were found in 16/27 children affected with mild PE (59.3%), 47/106 children exposed to severe PE (44.3%), 26/49 children exposed to early PE (53.1%), and 37/84 children affected with late PE (44.0%).

### 2.9. Increased Expression of miR-133a-3p in Children Descending from PE Pregnancies

**Figure 9:**
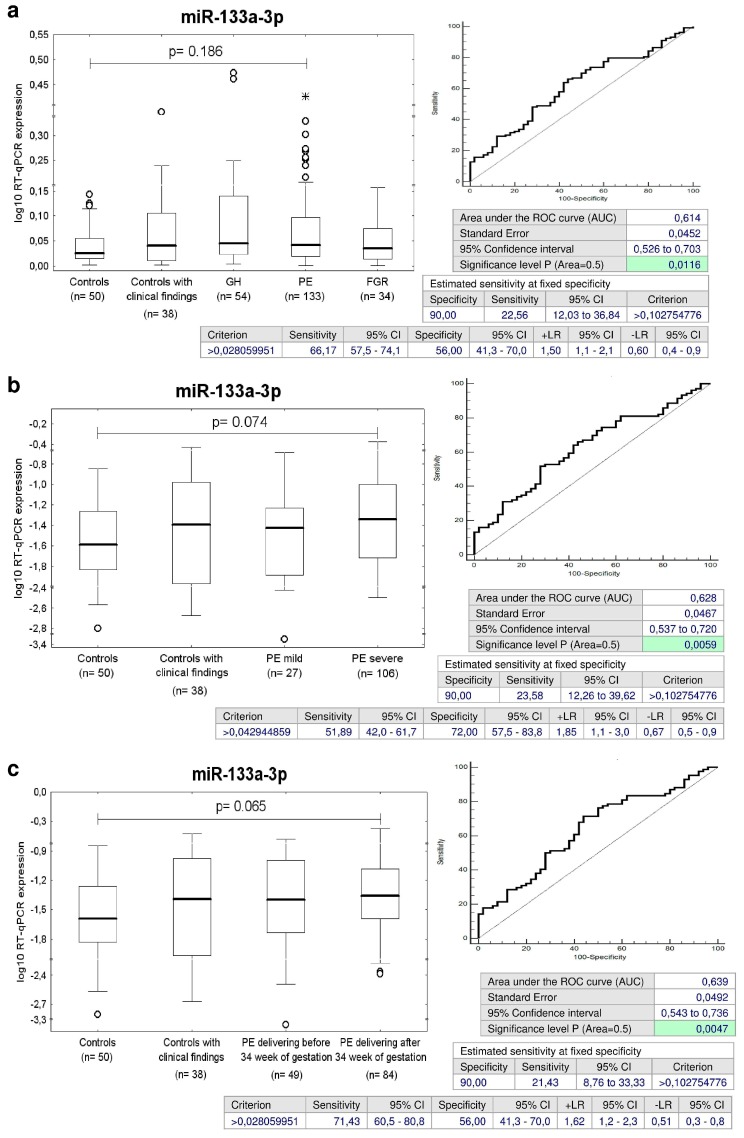

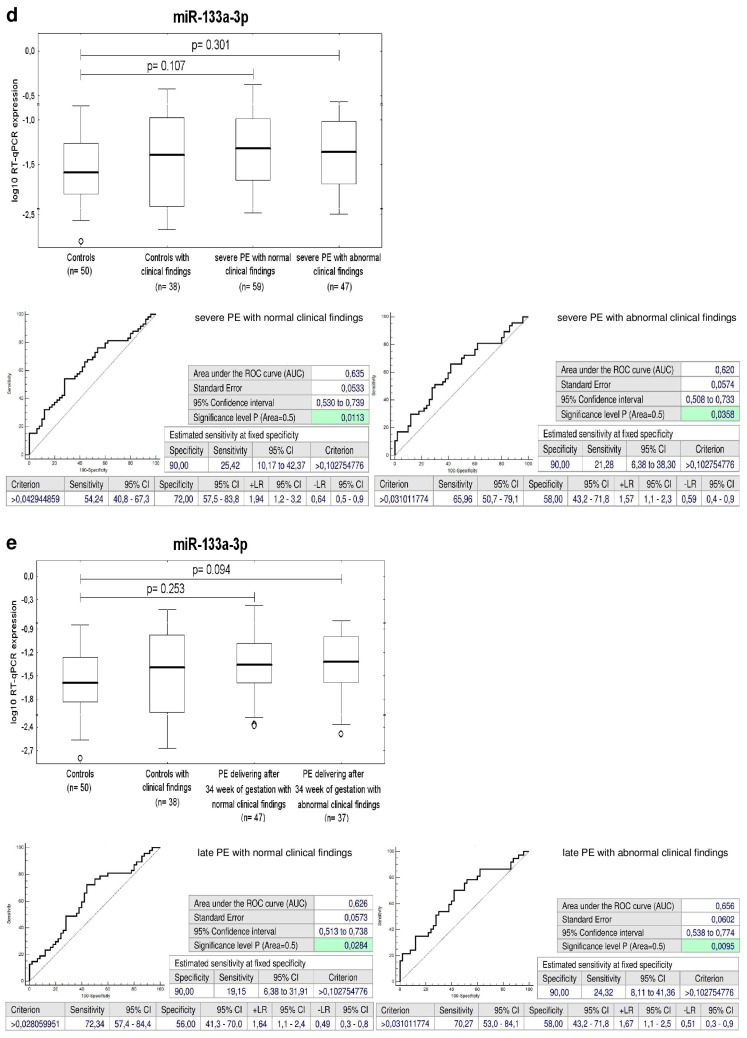

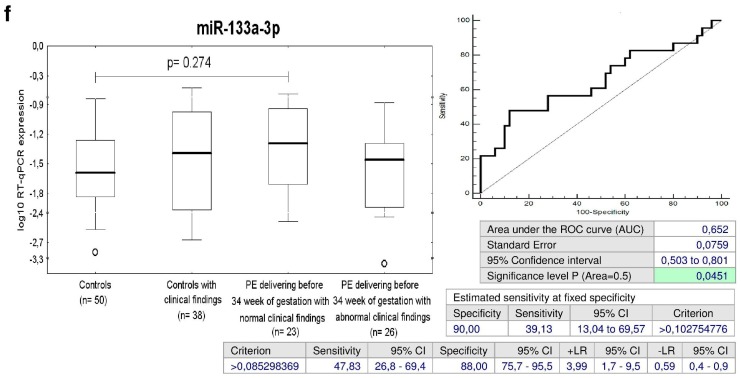
Increased expression of miR-133a-3p in children descending from PE pregnancies. (**a**–**c**) Increased expression of miR-133a-3p was observed in children descending from PE pregnancies regardless of the severity of the disease and delivery date, severe PE and late PE; (**d**,**e**) Increased expression of miR-133a-3p was found in children with both normal and abnormal postnatal clinical findings previously exposed to severe PE and late PE; (**f**) Increased expression of miR-133a-3p was found in children with normal postnatal clinical findings previously exposed to early PE.

The ROC curve analysis was able to identify up-regulated expression profile of miR-133a-3p in 22.56% children affected with PE regardless of the severity of the disease and the delivery date at 10.0% FPR (Figure 9a). 

Subsequently, miR-133a-3p differentiated between children affected with severe PE and controls with a sensitivity of 23.58% at a specificity of 90.0% (Figure 9b).

Parallel, miR-133a-3p was able to identify children exposed to late PE with a sensitivity of 21.43% at a specificity of 90.0% (Figure 9c).

In addition to that, the consecutive analysis showed that the accuracy of miR-133a-3p biomarker for severe PE affected children identified to have normal or abnormal clinical findings was 25.42% and 21.28% sensitivity at 10.0% FPR (Figure 9d).

Similarly, postnatal screening of miR-133a-3p was able to identify children with a history of late PE with either normal or abnormal clinical findings with a sensitivity of 19.15% and 24.32% at a specificity of 90.0% (Figure 9e). 

Parallel, ROC curve analysis of miR-133a-3p identified a significant proportion of children with normal clinical findings exposed to early PE during gestation (a sensitivity of 39.13% at 10.0% FPR) (Figure 9f).

### 2.10. Increased Expression of miR-1-3p, miR-20a-5p, miR-103a-3p, and miR-342-3p in Children with Abnormal Clinical Findings Descending from PE Pregnancies

**Figure 10:**
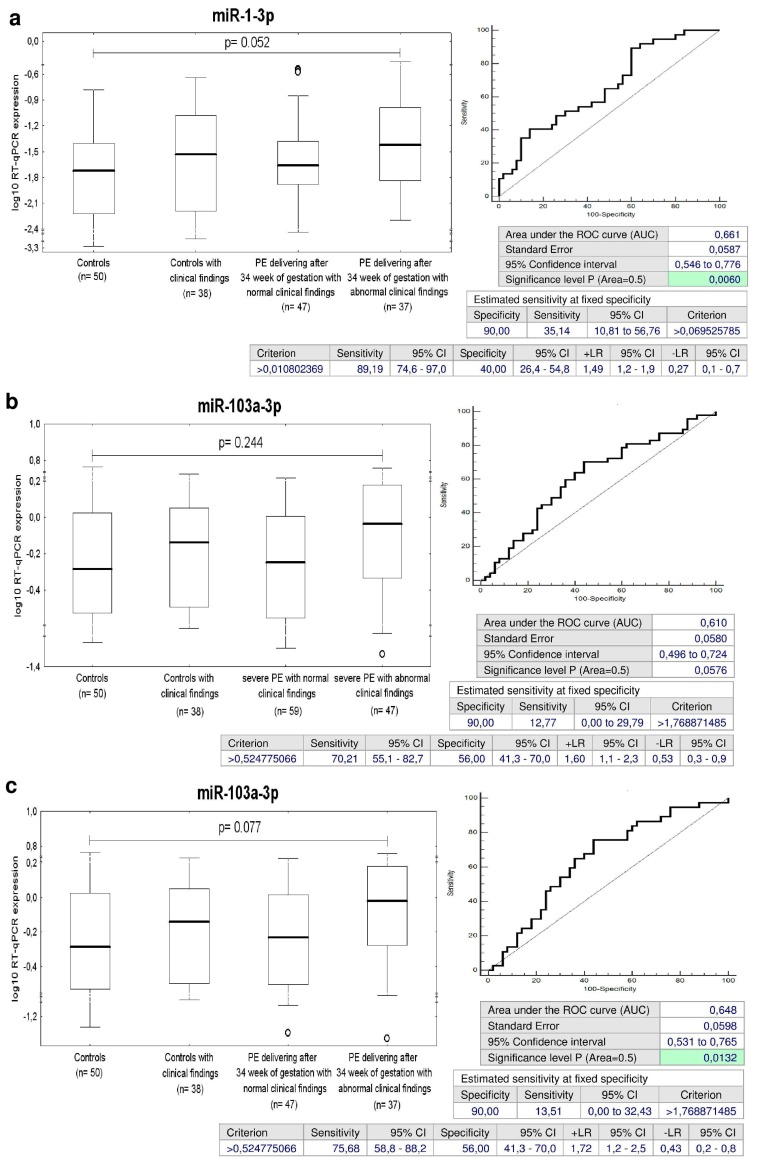

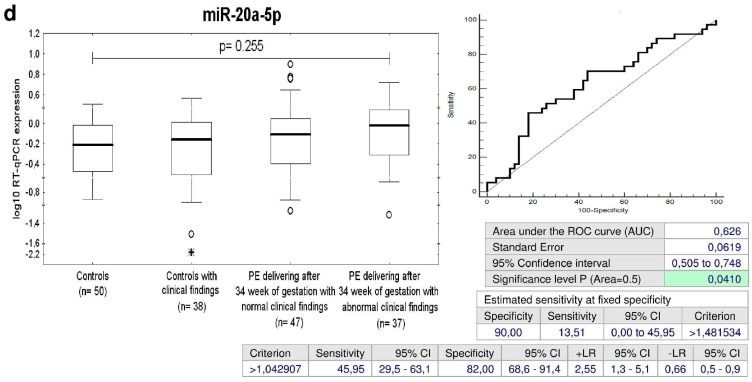
Postnatal microRNA expression profile in children with abnormal postnatal clinical findings descending from PE pregnancies. (**a**) Increased expression of miR-1-3p was found in children with abnormal postnatal clinical findings exposed to late PE; (**b**,**c**) Increased expression of miR-103a-3p was observed in children with abnormal postnatal clinical findings exposed to severe PE or late PE; (**d**) Increased expression of miR-20a-5p was found in children with abnormal postnatal clinical findings with a prior exposure to late PE.

The sensitivity at 10.0% FPR for miR-1-3p for children exposed to late PE was 27.38% and miR-1-3p showed even a higher performance for children with abnormal postnatal clinical findings that were exposed to late PE (a sensitivity of 35.14% at a specificity of 90.0%) (Figure 10a). 

Furthermore, at 10.0% FPR, miR-103a-3p was up-regulated in 12.77% and 13.51% children with abnormal clinical findings that were exposed to severe PE or late PE (Figure 10b,c).

In addition, miR-20a-5p was up-regulated in 13.51% children with abnormal clinical findings with a prior exposure to late PE (Figure 10d). 

**Figure 11:**
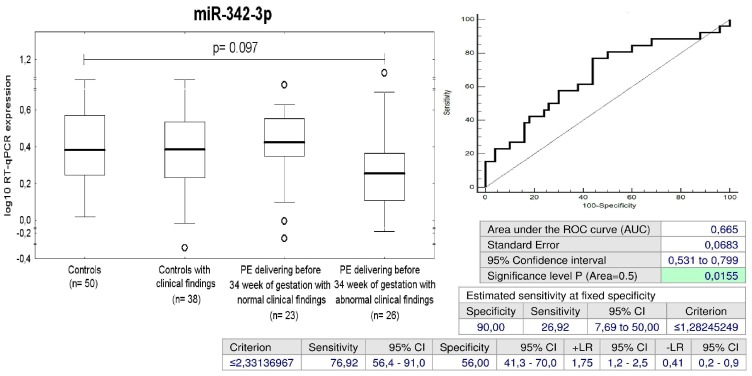
Decreased expression of miR-342-3p in children with abnormal postnatal clinical findings descending from early PE pregnancies.

MiR-342-3p represented the unique marker, which was able to differentiate between the group of children with abnormal clinical findings that were exposed to early PE and the controls (a sensitivity of 26.92% at 10.0% FPR) (Figure 11).

**Figure 12:**
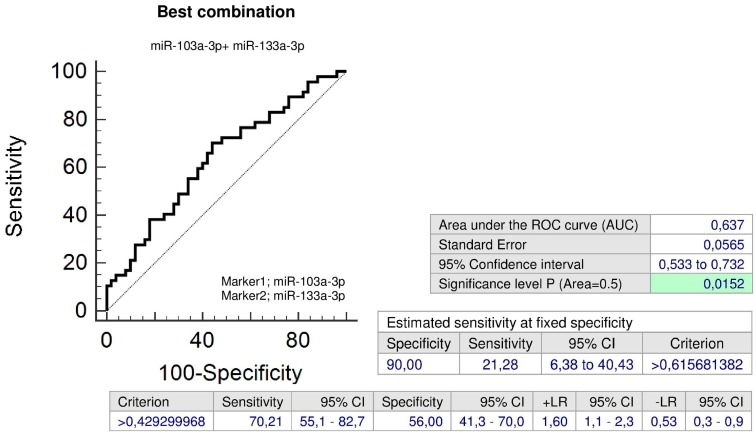
Combined postnatal screening of microRNAs in the identification of children with abnormal postnatal clinical findings descending from severe PE pregnancies. Postnatal combined screening of miR-103a-3p and miR-133a-3p showed the highest accuracy for the identification of children with abnormal clinical findings with a prior exposure to severe PE at a higher risk of later development of cardiovascular/cerebrovascular diseases.

The combination of miR-103a-3p and miR-133a-3p (AUC 0.637, *p* = 0.015, sensitivity 70.21%, specificity 56.0%, cut off > 0.429300) was superior over using only individual microRNA biomarkers, since it was able to identify at 10.0% FPR within the group of children with abnormal clinical findings previously exposed to severe PE 21.28% children with an increased cardiovascular risk (Figure 12).

**Figure 13:**
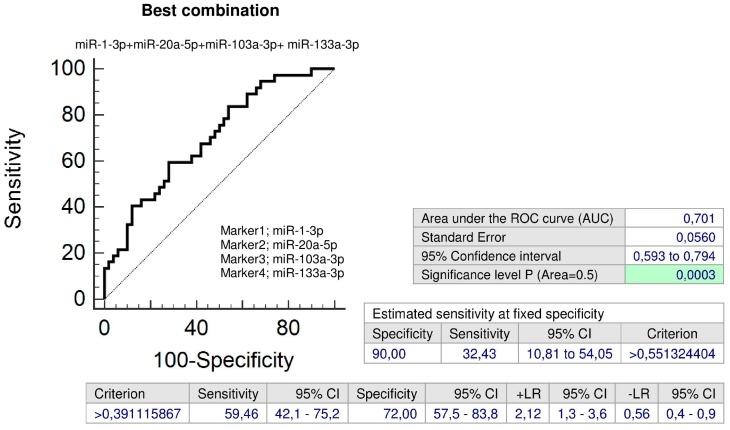
Combined postnatal screening of microRNAs in the identification of children with abnormal postnatal clinical findings descending from late PE pregnancies. Postnatal screening based on the combination of miR-1-3p, miR-20a-5p, miR-103a-3p, and miR-133a-3p showed the highest accuracy for the identification of children with abnormal clinical findings with a prior exposure to late PE at a higher risk of later development of cardiovascular/cerebrovascular diseases.

Parallel, combined screening of four microRNAs (miR-1-3p, miR-20a-5p, miR-103a-3p, and miR-133a-3p) showed the highest accuracy for children with abnormal clinical findings with a prior exposure to late PE (AUC 0.701, *p* < 0.001, sensitivity 59.46%, specificity 72.0%, cut off > 0.391116). It was able to identify 32.43% children with an increased cardiovascular risk at 10.0% FPR (Figure 13). 

### 2.11. Up-Regulation of miR-20b-5p in Children with Normal Clinical Findings Descending from Mild PE Pregnancies 

**Figure 14:**
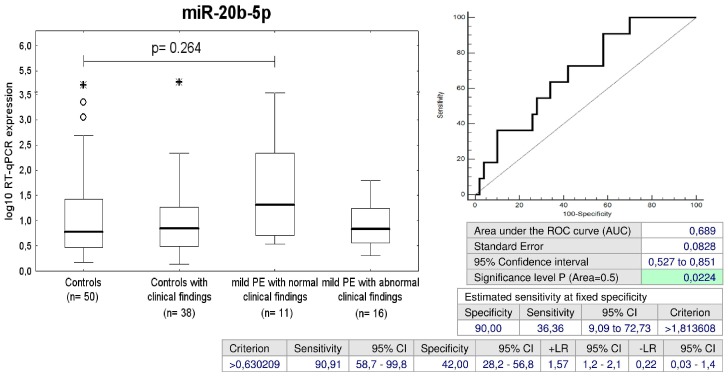
Increased expression of miR-20b-5p in children with normal postnatal clinical findings descending from mild PE pregnancies.

MiR-20b-5p was the only biomarker that differentiated between the group of children with normal clinical findings that were exposed to mild PE and the controls (a sensitivity of 36.36% at 10.0% FPR) (Figure 14). 

### 2.12. Dysregulation of Cardiovascular/Cerebrovascular Disease Associated microRNAs in Children Descending from FGR Pregnancies 

Overall, the group with abnormal postnatal clinical findings consisted of 22/34 children (64.7%) exposed to FGR (9 children already dispesarized in the department of pediatric cardiology, 9 children with abnormal echocardiographic findings (4 tricuspid valve regurgitation, 3 pulmonary valve regurgitation, 1 atrial septum defect, 6 foramen ovale apertum), 9 children with prehypertension/hypertension and 1 child with high BMI). Abnormal clinical findings were found in 7/13 children affected with early FGR (53.85%), and in 15/21 children exposed to late FGR (71.43%).

### 2.13. Increased Expression of miR-17-5p, miR-126-3p and miR-133a-3p in Children with Abnormal Clinical Findings Descending from FGR Pregnancies 

**Figure 15:**
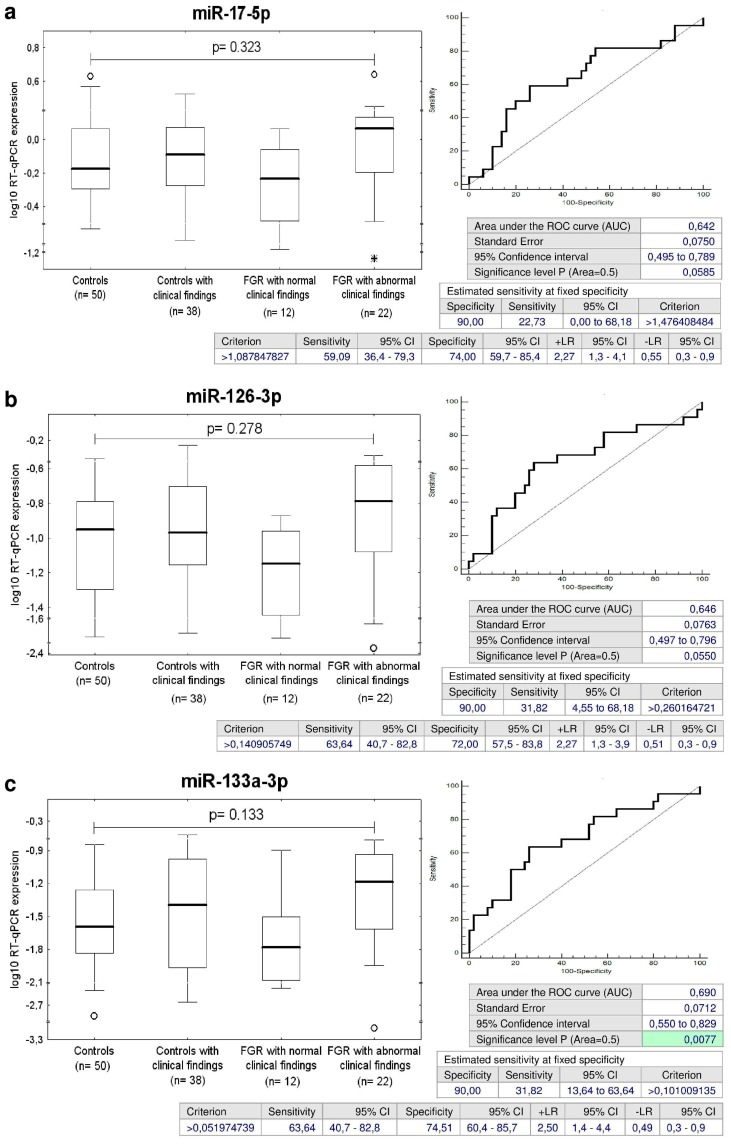
Postnatal microRNA expression profile in children with abnormal postnatal clinical findings descending from FGR pregnancies. (**a**–**c**) Increased expression of miR-17-5p, miR-126-3p, and miR-133a-3p was observed in children with abnormal postnatal clinical findings descending from FGR pregnancies.

MiR-17-5p, miR-126-3p, and miR-133a-3p differentiated between the group of children with abnormal clinical findings that were affected with FGR and the controls with a sensitivity of 22.73 %, 31.82% and 31.82% at a specificity of 90.0% (Figure 15a–c). 

**Figure 16:**
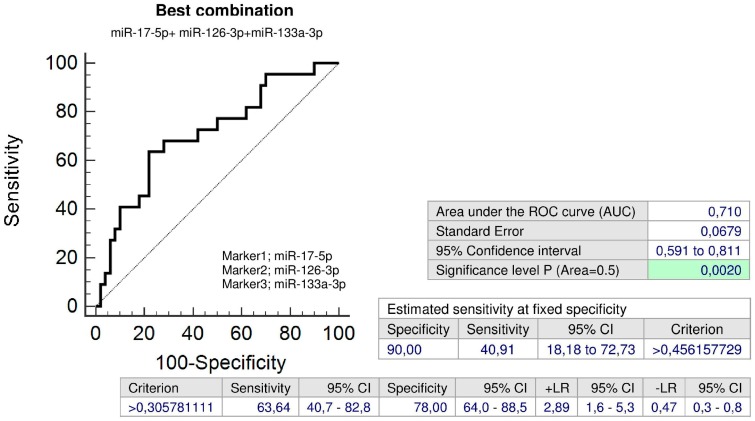
Combined postnatal screening of microRNAs in the identification of children with abnormal postnatal clinical findings descending from FGR pregnancies. Postnatal screening based on the combination of miR-17-5p, miR-126-3p and miR-133a-3p showed the highest accuracy for the identification of children with abnormal clinical findings with a prior exposure to FGR at a higher risk of later development of cardiovascular/cerebrovascular diseases.

The combination of all examined microRNAs (AUC 0.710, *p* = 0.002, sensitivity 63.64%, specificity 78.0%, cut off > 0.305781) was superior over using only individual microRNA biomarkers, since it was able to identify at 10.0% FPR within the group of children with abnormal clinical findings affected with FGR 40.91% children with an increased cardiovascular risk (Figure 16).

### 2.14. The Association between Postnatal Expression of miR-210-3p and the Severity of PE and/or FGR with regard to Doppler Ultrasonography Parameters

**Figure 17:**
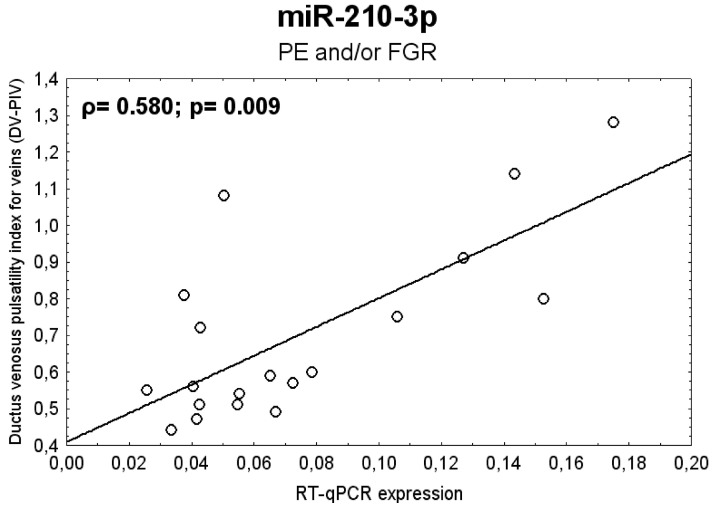
Association between postnatal miR-210-3p expression and the pulsatility index in the ductus venosus in PE and/or FGR patients. The pulsatility index in the ductus venosus showed a strong positive correlation with miR-210-3p gene expression in patients with a history of PE and/or FGR.

The index of pulsatility in the ductus venosus (DV) showed a strong positive correlation with miR-210-3p gene expression in children with a history of PE and/or FGR (Figure 17). That means that children with prior findings of DV dilatation indicating poor outcome in severe FGR demonstrated increased postpartum levels of miR-210-3p. 

The results of aberrant expression profile of microRNAs in children descending from pregnancy-related complications are summarized in Table 1.

### 2.15. The Effect of Children Age on Particular microRNA Expression in Children Descending from Normal and Complicated Pregnancies

The effect of children age on particular microRNA expression in children descending from normal and complicated pregnancies is discussed in supplementary file (Figures S1–S4).

## 3. Discussion

Cardiovascular/cerebrovascular disease associated microRNA expression profile was assessed in children at the age of 3 to 11 years. Subsequently, epigenetic profile was compared between groups descending from pregnancies that had normal course of gestation or were complicated by GH, PE, and FGR, and correlated with the severity of the disease with regard to clinical signs (mild versus severe preeclampsia), dates of delivery (< and >34 weeks in case of PE, < and >32 weeks in case of FGR, respectively), and Doppler ultrasound parameters.

Initially, we collected the anamnesis at children and parents, performed medical examination (BP measurement, and BMI assessment) and utilized Doppler echocardiography to check the heart’s anatomy and function and diagnose or rule out cardiac problems in the studied cohorts. That is the reason why the control group of children after normal pregnancies consisted just of those children who had normal BP, normal BMI, normal reference values of echocardiographic measurements and no cardiac findings in anamnesis. The second group of offspring born out of normal pregnancies consisted of children either already dispensarized in the department of pediatric cardiology, or those ones who were indicated during our medical examination to have valve problems and heart defects, abnormal BP or BMI. In general, the expression profile of microRNAs was equal between these two groups of children descending from uncomplicated pregnancies with an exception of miR-21-5p, which was up-regulated in a proportion of children with abnormal clinical findings (28.95%). According to our opinion, this group of children would benefit from dispensarization and implementation of primary prevention strategies, since it may be at higher risk of later development of cardiovascular diseases. 

Since we identified a large proportion of children with abnormal clinical findings within groups descending from complicated pregnancies (57.41% children with a prior exposure to GH, 47.37% children exposed to PE, and 64.7% children affected with FGR), we analyzed also microRNA gene expression in relation to both aspects, the current presence or absence of cardiovascular risk factors (overweight/obesity and/or prehypertension/hypertension) and cardiovascular complications (valve problems and heart defects) and the previous occurrence of pregnancy-related complications. 

As expected, the expression profile of microRNAs differed between children with a history of complicated pregnancies (GH, PE, and FGR) and controls. With respect to particular pregnancy-related complication subtypes, abnormal expression profile of multiple microRNAs was found in children affected with GH (15/29 studied microRNAs: miR-1-3p, miR-17-5p, miR-20a-5p, miR-21-5p, miR-23a-3p, miR-26a-5p, miR-29a-3p, miR-103a-3p, miR-125b-5p, miR-126-3p, miR-133a-3p, miR-146a-5p, miR-181a-5p, miR-195-5p, and miR-342-3p), clinically established PE (6/29 studied microRNAs: miR-1-3p, miR-20a-5p, miR-20b-5p, miR-103a-3p, miR-133a-3p, miR-342-3p) and FGR (3/29 studied microRNAs: miR-17-5p, miR-126-3p, miR-133a-3p). 

Interestingly, a set of microRNAs associated with cardiovascular/cerebrovascular diseases (miR-1-3p, miR-17-5p, miR-29a-3p, miR-126-3p, miR-133a-3p, miR-146a-5p, and miR-181a-5p) was dysregulated in both groups of children with a prior exposure to GH regardless of the occurrence of postnatal clinical findings. Seven additional microRNAs (miR-21-5p, miR-23a-3p, miR-26a-5p, miR-103a-3p, miR-125b-5p, miR-195-5p, and miR-342-3p) were observed to be dysregulated in children affected with GH with normal postnatal clinical findings. In addition, higher expression levels of miR-20a-5p were detected in children with a prior exposure to GH, who were found to have abnormal clinical findings.

With regard to miR-133a-3p, a group of children exposed to PE produced similar findings to the group of children affected with GH. MiR-133a-3p up-regulation appeared in 37.04% and 22.56% children descending from GH or preeclamptic pregnancies irrespective of the postnatal clinical findings. Nevertheless, miR-133a-3p up-regulation was detected mainly in a group of children affected with severe PE (23.58%) and late PE (21.43%). On the other hand, up-regulation of miR-133a-3p appeared only in those children affected with FGR that were identified to have abnormal clinical findings (31.82%). 

This study showed similar findings to our previous study [44], where we observed increased expression of miR-133a-3p in umbilical cord blood from patients with PE and/or FGR that had decreased values of the CPR indicating a protective reaction of the fetus against hypoxia. We suggest that the increased expression of miR-133a-3p found in a proportion of children previously exposed to GH, PE and/or FGR may be a long-term consequence of pregnancy-related complications. We suppose that up-regulation of miR-133a-3p may have rather compensatory than harmful effect, but postnatal miR-133a-3p screening may stratify a risky group of children predisposed to later cardiovascular disease development.

Interestingly, the data resulting from our previous studies [44,45] indicated that miR-1-3p was significantly up-regulated in placental tissues or umbilical cord blood of PE and/or FGR patients with an abnormal index of pulsatility in the umbilical artery or the signs of centralization of the fetal circulation, or in late PE, apparently as a consequence of an incapacity of maternal cardiovascular system to deal with the demands of an advanced pregnancy. That’s why the increased expression of miR-1-3p present in circulation of children born out of pregnancies complicated with GH or late PE may also be associated with previous occurrence of hypertensive disorders of pregnancy. A large proportion of children with an up-regulated miR-1-3p profile with a prior exposure to GH (47.83% children with normal clinical findings and 45.16% children with abnormal clinical findings) or late PE (35.14% children with abnormal clinical findings) was identified. We suppose that children descending from complicated pregnancies with aberrant expression profile of miR-1-3p are at a higher risk of later onset of cardiovascular diseases, and should be carefully monitored in the long term. 

This study demonstrated that the dysregulation of at least two microRNAs (miR-1-3p and miR-133a-3p) caused by pregnancy complications in placental tissues and/or umbilical cord blood is present as well in circulation of children with hindsight (3 to 11 years after the birth) after the exposure to GH, PE, or FGR. It is obvious that changes in epigenome induced by pregnancy complications in placental tissue and umbilical cord blood may cause later development of cardiovascular and cerebrovascular diseases in offspring. An impact of the environment on early life epigenetic programming might support the phenomena known as developmental programming and explain the developmental origins of diseases. It is well known that complex diseases, such as diabetes, obesity, and heart disease, result from the interaction between genetic and environmental factors. Alternatively, other explanations to the aberrant microRNA expression in pregnancy-related complications may be taken into consideration. For instance, it may be that complicated pregnancies induce changes in proportional representation of any of cell subpopulations including endothelial stem cells and immune cells, which may be the cause of different microRNA expression. 

However, it is also evident that epigenetic profiles of other microRNAs have been changing with time by force of various circumstances, since several microRNAs which were up-regulated in children exposed to GH (miR-17-5p, miR-20a-5p, miR-21-5p, miR-23a-3p, miR-29a-3p, miR-146a-5p, and miR-181a-5p), PE (miR-20a-5p, and miR-20b-5p) or FGR (miR-17-5p) were not observed to be dysregulated in placental tissues and/or umbilical cord blood during the clinical manifestation of pregnancy-related complications [44,45].

In parallel, several microRNAs which were up-regulated in children exposed to GH (miR-26a-5p, miR-103a-3p, miR-125b-5p, miR-126-3p, miR-195-5p, and miR-342-3p), PE (miR-103a-3p, and miR-342-3p) or FGR (miR-126-3p) were observed to be down-regulated in placental tissues and/or umbilical cord blood during the clinical manifestation of pregnancy-related complications [44,45].

However, existing data suggests that these microRNAs play a role in the pathogenesis of cardiovascular/cerebrovascular diseases (Table 2) [21,23,25,29,39,46,47,48,49,50,51,52,53,54,55,56,57,58,59,60,61,62,63,64,65,66,67,68,69,70,71,72,73,74,75,76,77,78,79,80,81,82,83,84,85,86,87,88,89,90,91,92,93,94,95,96,97,98,99,100,101,102,103,104,105,106,107,108,109,110,111,112,113,114,115,116,117,118,119,120,121,122,123,124,125,126,127,128,129,130,131,132,133,134,135,136,137,138].

MiR-1-3p is from the precursors for miR-1-1 and miR-1-2 encoded by distinct genes located on chromosome 20q13.3 and on chromosome 18q11.2 [46]. MiR-1 is abundantly expressed in cardiac and skeletal muscles, especially in myocardium. Extracellular miR-1 levels are significantly increased in patients with acute MI and highly correlate with circulating troponin T, a reliable marker of cardiac damage [47]. MiR-1 also represents a promising therapeutic target in treatment of cardiovascular diseases, heart ischemia, and post-MI complications. Inhibition of miR-1 with antisense oligonucleotides is cardioprotective, since it leads to reduction of apoptosis, increase of resistance to oxidative stress, and attenuation of spontaneous arrhythmogenic oscillations [48,49,50]. 

A large proportion of children with an up-regulated miR-1-3p profiles with a prior exposure to GH (47.83% children with normal clinical findings and 45.16% children with abnormal clinical findings) or late PE (35.14% children with abnormal clinical findings) was identified. We suppose that children descending from complicated pregnancies with aberrant expression profiles of miR-1-3p are at a higher risk of later onset of cardiovascular diseases, and should be carefully monitored in the long term. 

MiR-17-5p, a member of miR-17-92 cluster, located on the human chromosome 13q31.3 [51,52], is overexpressed in endothelial cells and lowly expressed in vascular smooth muscle cells [53]. MiR-17p~92 microRNAs were found to play a major role in cardiac development, since the hearts of miR-17p~92 deficient mutant embryos presented a clear ventricular septal defect [54]. Moreover, multiple studies have also confirmed the involvement of miR-17-5p in regulating ischemia/reperfusion-induced cardiac injury (I/R-I). Up-regulation of miR-17-5p has been reported to promote apoptosis induced by oxidative stress via targeting Stat3 in in vivo I/R-I mouse model and in vitro cellular model of oxidative stress induced by H_2_0_2_ [55]. The inhibition of miR-17-5p by its specific inhibitors preserved cell survival and rescued cell death in both in in vivo I/R-I mouse and in vitro cellular oxidative stress models [55] and improved cardiac function after acute myocardial infarction via weakening of apoptosis in endothelial cells in SD rat model [56]. Kaucsar et al. reported that miR-17-5p was activated during kidney ischemia-reperfusion injury in mice [57]. In humans, circulating miR-17-5p represents one of the potential up-regulated biomarkers for diffuse myocardial fibrosis in hypertrophic cardiomyopathy [58], in patients with acute ischemic stroke [59], and for the severity of coronary artery disease [60]. Our data showed that miR-17-5p was overexpressed in a proportion of children with a prior exposure to GH, regardless of normal or abnormal clinical findings (30.43% versus 29.03%), and in children previously affected with FGR, however only with abnormal clinical findings (22.73%). In view of the fact that the overexpression of miR-17-5p is associated with a high cardiovascular risk, we suppose that children with aberrant expression profile of miR-17-5p need to be stratified as soon as possible to prevent them from later cardiovascular disease development. 

MiR-20a-5p belongs to the miR-17 family and is also transcribed from the miR-17-92 cluster located on the human chromosome 13q31.3 [61]. MiR-20a is involved with inflammatory signaling in pulmonary hypertension [62]. Intraperitoneal injections of antagomiR-20a significantly down-regulated the expression levels of miR-20a-5p and restored functional levels of bone morphogenetic protein receptor type 2 (BMPR2) in pulmonary arteries in hypoxia-induced pulmonary hypertension mouse model [62]. A previous pilot study of Zhu et al. demonstrated increased plasma levels of miR-17-5p and miR-20a-5p also in patients diagnosed with gestational diabetes mellitus (GDM) at 16–19 weeks of gestation [63]. Our results support the involvement of miR-20a-5p in pathogenesis of pathologies enhancing a cardiovascular risk, since overexpression of miR-20a-5p was identified only in a proportion of children with abnormal clinical findings that were previously exposed to GH (25.81%) or preeclampsia terminated after 34 weeks of gestation (13.51%). The early identification of this risky group of children may improve their future cardiovascular health.

MiR-20b-5p also belongs to the miR-17 family, however is transcribed from the miR-106a-363 cluster located on chromosome Xq26.2 [61]. Recent study showed that increased plasmatic levels of miR-20b served as one of selected biomarkers in hypertension-induced heart failure in adult male Dahl salt-sensitive rats [64]. In addition, higher levels of miR-20b-5p were found in second trimester maternal sera of pregnancies with small-for-gestational age fetuses when compared with appropriate-for-gestational age fetuses [65]. In our study, mir-20b-5p represented a unique biomarker that was up-regulated in a proportion of children with contemporary normal clinical findings that were previously affected with mild preeclampsia (36.36%). Due to the lack of information on aberrant expression profile of miR-20b-5p in cardiovascular diseases, its role in children descending from complicated pregnancies remains for us unclear.

MiR-21-5p and miR-21-3p are encoded by the gene located on chromosome 17q23.2 [66] and mediate the homeostasis of the cardiovascular system [67]. Overexpression of miR-21 promotes cardiac fibrosis and development of heart failure with preserved left ventricular ejection fraction. Inhibition of miR-21 by miR-21 antagonists led to amelioration of cardiac atrophy and cardiac fibrosis [68]. MiR-21 is upregulated by HIF-1α under hypoxia in cardiomyocytes and silencing of HIF-1α and inhibition of miR-21 increase the apoptosis of hypoxic cardiomyocytes [69]. Extracellular miR-21 can be used as a biomarker for the diagnosis and prognosis of heart failure. Serum levels of miR-21 were higher in patients with heart failure than in controls, and correlated with ejection fraction and brain natriuretic peptide [70]. In our study miR-21-5p was up-regulated in a proportion of children descending from normal pregnancies with abnormal clinical findings (28.95%) and in children descending from GH pregnancies with normal clinical findings (39.13%). According to our opinion, this group of children would benefit from dispensarization and implementation of primary prevention strategies, since it may be at higher risk of later development of cardiovascular diseases. 

MiR-23a, encoded by a gene located at chromosome 19p13.12, has two mature microRNAs: miR-23a-5p and miR-23a-3p. MiR-23a regulates cardiomyocyte apoptosis, a key pathogenesis factor of heart failure, by targeting manganese superoxide dismutase [71] and the vasculogenesis of coronary artery disease via targeting epidermal growth factor receptor [72]. Extracellular miR-23a may be a new biomarker for coronary artery disease, since increased levels of miR-23a may be used to predict the presence and severity of coronary lesions in patients with coronary artery disease [72]. MiR-23a-3p also suppressed oxidative stress injury in a mouse model of focal cerebral ischemia-reperfusion [73]. Since our study demonstrated overexpression of miR-23a-3p in a proportion of children with normal clinical findings born of GH complicated pregnancies only (34.78%), we suppose that compensatory effect of miR-23a-3p may appear more likely in these children to normalize cardiomyocyte state and vasculogenesis.

MiR-26a-5p is produced by miR-26a-1 and miR-26a-2, whose genes are located on chromosomes 3p22.2 and 12q14.1 [74]. MiR-26a-5p was demonstrated to regulate the autophagy in cardiac fibroblasts by targeting a key component of autophagy pathway, ULK1 (unc-51 like autophagy activating kinase 1). Overexpression of miR-26a-5p reduces the expression of ULK1 and decreases the activation of LC3-I to LC3-II (microtubule-associated protein 1 light chain) participating in the formation of autophagosome membranes during autophagy [75]. Autophagy plays a protective role in heart failure and cardiac hypertrophy by removing damaged proteins [65]. Nevertheless, in some cases inhibited autophagy may also improve cardiac function [75]. Since our study showed overexpression of miR-26a-5p in a proportion of children with normal clinical findings that were affected with GH (21.74%), we suggest that this phenomenon may have protective effect against potential development of cardiac fibrosis.

MiR-29a-3p, a member of miR-29 family, is encoded by a gene located on chromosome 7q32.3. Antagomirs against miR-29a significantly increased Mcl-2 expression and significantly reduced myocardial infarct size and apoptosis in rat hearts subjected to ischaemia-reperfusion injury [76]. Increased expression of miR-29a-3p was also observed in cardiac cachexia, a common complication of heart failure, in Wistar rat models [77]. In patients with atrial fibrillation, overexpression of miR-29a-3p found in the biopsies collected from the right atrial appendage during the general surgical procedure was associated with underexpression of calcium voltage-gated channel subunit alpha 1C (CACNA1C) [78]. Circulating miR-29a-3p represents one of potential up-regulated biomarkers for diffuse myocardial fibrosis in hypertrophic cardiomyopathy [58]. Previous pilot study of Zhu et al. demonstrated increased plasma levels of miR-29a-3p in GDM patients at 16–19 weeks of gestation [63]. Serum mir-29a-3p levels were also shown to be elevated in patients with recent diagnosis of type 2 diabetes mellitus (T2DM) [23]. Moreover, overexpression of miR-29a-3p was found in resistance arterioles obtained by biopsy from T2DM patients [79]. Our study indicated the presence of overexpression of miR-29a-3p in both groups of children with normal and abnormal clinical findings descending from pregnancies complicated by GH (39.13% versus 32.26%). We believe that this group of children is endangered by later development of cardiovascular diseases and would benefit from early prevention programs to decrease a cardiovascular risk. 

MiR-103 and miR-107 are paralogous microRNAs binding the same target sites. MiR-103 is encoded by two different genes located on chromosomes 5q34 and 20p13 [80]. Both genes generate miR-103a-3p. MiR-103/107 regulate programmed necrosis and myocardial ischemia/reperfusion injury via targeting FADD (Fas-associated protein with death domain). Both miR-103 and miR-107 are overexpressed in the ischemic zone of ischemic heart. Plasma miR-103a concentration is also significantly elevated in patients with high blood pressure and acute myocardial infarction [81]. Injection of miR-103/107 antagomir led to a reduction of FADD and induced a reduction in the myocardial necrosis and myocardial infarct sizes [82]. MiR-103/107 is also involved in hypoxia-induced pulmonary hypertension through hypoxia-induced proliferation of pulmonary arterial smooth muscle cells via targeting HIF-1β. Nevertheless, in this model miR-103/107 induced overexpression led to inhibition of hypoxia induced pulmonary arterial smooth muscle cell proliferation and hypoxia-induced pulmonary hypertension [83]. In addition, miR-103/107 play the central importance in regulation of insulin sensitivity. Overexpression of miR-103/107 is present in obese mice and silencing of miR-103/miR-107 leads to the improvement of glucose homeostasis and insulin sensitivity [84]. Our data are similar to findings of Huang et al. [81] and Trajkovski et al. [84]. Since we identified overexpression of miR-103a-3p in a proportion of children with abnormal clinical findings who were exposed to severe preeclampsia (12.77%) and or preeclampsia developing after 34 weeks of gestation (13.51%), we consider up-regulation of miR-103-3p in circulation of children as a highly risky feature of potential development of cardiovascular diseases.

There are two paralogs, miR-125b-1 on chromosome 11q24.1 and miR-125b-2 on chromosome 21q21.1, both producing miR-125b-5p [85]. A set of upregulated circulating microRNAs including miR-125b-5p was identified to be associated with acute ischemic stroke [86] and acute myocardial infarction [87]. Overexpression of miR-125b-5p was shown to protect endothelial cells from apoptosis under oxidative stress via negative regulation of SMAD4 (SMAD family member 4) [88]. In addition, miR-125b-5p was identified as an ischemic stress-responsive protector against cardiomyocyte apoptosis. Cardiomyocytes overexpressing miR-125b-5p have increased prosurvival signaling and protected the heart from acute myocardial infarction by repressing pro-apoptotic bak1 and klf13 [89]. Our results are consistent with the data of Wei et al. [88] and Bayoumi et al. [89], since we observed overexpression of miR-125b-5p in a significant proportion of children with normal clinical findings that were previously affected with GH (47.83%). We suggest that a protective effect of miR-125-5p may be present just in children previously exposed to minor pregnancy-related complications, while in children exposed to severe pregnancy-related complications it apparently vanished. 

MiR-126, producing miR-126-3p, is an intronic microRNA located in intron 7 of EGFL7 (epidermal growth factor-like protein 7) gene on chromosome 9q34.3 [90]. MiR-126 regulates endothelial expression of vascular cell adhesion molecule 1 (VCAM-1) and controls vascular inflammation. Overexpression of miR-126 decreases VCAM-1 expression and in opposite transfection of endothelial cells with miR-126 antagomirs increases TNFα-stimulated VCAM-1 expression [21]. MiR-126-3p was down-regulated in the sera derived from patients with acute myocardial infarction [91] and in the plasma of type 2 diabetes patients [92]. Recent experiments also demonstrated that intercellular transfer of miR-126-3p by endothelial microparticles reduced vascular smooth muscle cell proliferation and limited neointima formation via inhibition of LRP6 (LDL receptor related protein 6) [93]. It seems that compensatory role of miR-126-3p also appears in a proportion of children with both normal or abnormal clinical findings with a prior exposure to GH (39.13% versus 22.58%) or FGR (31.82%), most likely with the aim to induce suppression of cytokine activated endothelial cells and vascular smooth muscle cell proliferation.

MiR-133a-3p belongs to the miR-133 family. MiR-133a-1 gene is located on chromosome 18q11.2 and miR-133a-2 gene on chromosome 20q13.33, respectively. Both genes produce miR-133a-3p [94]. MiR-133 was down-regulated with hypertrophy [95], heart failure [96], and down-regulation of miR-133 also contributed to arrhythmogenesis in the hypertrophic and failing hearts. Induction of overexpression of miR-133 reduced hypertrophy as well as lead to correction of conduction abnormalities [97,98]. Furthermore, circulating miR-133a-3p provides one of up-regulated candidates as potential biomarkers for diffuse myocardial fibrosis in hypertrophic cardiomyopathy [58] and coronary artery calcification [99]. We suggest that the overexpression of miR-133a-3p observed in our study in a proportion of children previously exposed to GH, PE and/or FGR may be a long-term consequence of pregnancy-related complications and we believe that postnatal miR-133a-3p screening may stratify a risky group of children predisposed to later cardiovascular disease development.

The miR-146 family consists of 2 members, with nearly identical sequences, miR-146a-5p and miR-146b-5p [100]. MIR146A gene was found within a larger long noncoding RNA host gene, MIR3142HG, on chromosome 5q33.3 [101]. MiR-146a-5p is actively involved in multiple oncological processes such as antitumor immune suppression, metastasis, and angiogenesis [102]. MiR-146a-5p is an anti-inflammatory microRNA, since it functions as a negative regulator of inflammation by targeting interleukin-1 receptor-associated kinase 1 (IRAK1) and tumor necrosis factor (TNF) receptor associated factor 6 (TRAF6), resulting in inhibition of NF-κB activation [103]. Furthermore, miR-146a is one of the microRNAs that is most sensitive to hypoxia. It seems that miR-146a overexpression can protect the myocardium from I/R damage. Lentivirus expressing miR-146a transfected into mouse hearts decreased I/R-induced myocardial infarct size and prevented I/R-induced decreases in ejection fraction and fractional shortening [104]. Increased circulating plasma levels of miR-146a also correlated with the severity of coronary atherosclerosis in patients with subclinical hypothyroidism and have been suggested as good predictor for coronary heart disease development among individuals with elevated TSH levels [105]. Nevertheless, a significant reduction of miR-146a expression was observed in acute ischemic stroke. A down-regulation of miR-146a was suggested to be a self-protective response of the brain against the consequences of acute cerebral ischemia injury via the up-regulation of Fbxl10 expression, which protects neurons from ischemic death [103]. Since we observed the overexpression of miR-146a-5p in both groups of children with normal and abnormal clinical findings, whose pregnancies were complicated by GH only (26.09% versus 12.9%), we believe that a protective role of miR-146a-5p is applied at least at a lesser group of children with the aim to reduce inflammation and its negative consequences. 

MiR-181a and miR-181b cluster together on chromosomes 1q32.1 and 9q33.3. MiR-181a generates several mature microRNAs involving miR-181a-5p, miR-181a-3p and miR-181a2-3p [106]. The miR-181 family plays a key role in both acute and chronic inflammatory disease states such as atherosclerosis, type 2 diabetes, and obesity [106]. Nevertheless, contradictory data are reported concerning expression levels of miR-181a in various pathological conditions. Decreased expression of miR-181a observed in monocytes of obese patients was associated with the metabolic syndrome and coronary artery disease [107]. However, another study reported that overexpression of miR-181a-5p in adipocytes upregulated insulin-stimulated AKT activation and reduced TNFα-induced insulin resistance [108]. Moreover, increased hepatic miR-181a impaired glucose and lipid homeostasis by silencing sirtuin 1 in non-alcoholic fatty liver disease [109]. Several contradictory data are also reported concerning serum levels of miR-181a in diabetic patients. While serum levels of miR-181a-5p were decreased in obese and diabetic patients [108], circulating levels of miR-181a were increased in type 1 diabetic children and adolescents [110]. Nevertheless, circulating miR-181a-5p levels were increased in patients with ischaemic stroke, transient ischaemic attack and acute myocardial infarction [111,112]. Our study revealed overexpression of miR-181a-5p in a proportion of children with both normal and abnormal clinical findings descending from GH pregnancies (39.13% and 25.81%). In view of the inconsistency between studies, the role of miR-181a-5p is not clear. Nevertheless, we believe that increased levels of miR-181a-5p in whole peripheral blood of children previously exposed to GH may predispose to later development of cardiovascular diseases.

MiR-195 gene is located on the chromosome 17p13.1 and generates two microRNAs, miR-195-5p and miR-195-3p [113]. MiR-195 is increasing in cardiac hypertrophy, and cardiac miR-195 overexpression results in heart failure [29,114]. MiR-195 is also a powerful regulator of the aortic extracellular matrix. Administration of miR-195 antagomirs led to significant elevation of elastin and collagens in the murine aorta, but has no effect on survival and aortic diameter size. Nevertheless, in plasma samples an inverse correlation between miR-195 and the presence of abdominal aortic aneurysms and aortic diameter was observed. Surprisingly, miR-195 plasma levels were decreased in abdominal aortic aneurysms [115]. On the other hand, aortic stenosis caused by leaflet calcification of the bicuspid aortic valve was associated with down-regulation of miR-195 [116]. The mechanism of miR-195 action is not completely understood. Since we observed overexpression of miR-195-5p in a proportion of children with normal clinical findings previously affected with GH only (34.78%), we suggest that overexpression of miR-195-5p may have rather protective role than harmful effect.

MiR-210-3p, encoded by a gene located on chromosome 11p15.5, is the most prominent microRNA consistently stimulated under hypoxic conditions. A significant increase of mir-210 levels was detected in placentas of women with preeclampsia, a condition that is characterized by hypoxia resulting from inadequate blood supply to the placenta [39]. Several microRNAs involving miR-210 were reported to be involved in atherosclerotic plaque formation through the regulation of endothelial apoptosis [25,117]. Nevertheless, some authors reported that up-regulated levels of miR-210 positively correlated with the level of endothelial cell apoptosis [117], while the others found that miR-210 blockage only led to induction of endothelial cell apoptosis and cell death in hypoxia [128]. Another study showed that up-regulation of miR-210 had cytoprotective effects, mainly in cardiomyocytes [119] and the skeletal muscle [118]. Increased serum levels of miR-210 may indicate early stages of atherosclerosis obliterans [120] and heart failure [121]. MiR-210 also regulates angiogenesis in response to ischemic injury to the brain. Overexpression of miR-210 may activate the Notch signaling pathway, which probably contributes to angiogenesis after cerebral ischemia [122]. In view of the fact that we observed a strong positive correlation between gene expression of miR-210-3p in whole peripheral blood of children with a history of preeclampsia and/or FGR and the pulsatility index in the ductus venosus, we believe that children descending from preeclampsia and/or FGR affected pregnancies who had high postnatal levels of miR-210-3p in their circulation represent a risky group, that is endangered by the onset of cardiovascular and cerebrovascular diseases in the future, since increased PI in the ductus venosus is a sign of a poor perinatological outcome.

MiR-342-3p is encoded by a gene located on chromosome 14q32.2. MiR-342-3p is considered as an obesity-associated microRNA, since it positively regulates adipogenesis of adipose-derived mesenchymal stem cells by suppressing CtBP2 and releasing the key adipogenic regulator C/EBPα from CtBP2 binding [123]. A set of up-regulated microRNAs expressed in peripheral blood mononuclear cells involving mir-342-3p was shared among patients with type 1 diabetes mellitus, type 2 diabetes mellitus and gestational diabetes mellitus [124]. Urinary exosomal miR-342 was also expressed at significantly elevated levels in type 2 diabetes mellitus patients [125]. However, miR-342-3p expression was significantly reduced in endothelial cells isolated from lung and heart tissues of type 2 diabetes mellitus mice and this inhibition blocked vasculogenesis in vivo by repressing endothelial proliferation and migration [126]. Decreased expression of miR-342 was also observed in T regulatory cells of patients with type 1 diabetes mellitus [127]. Moreover, plasma miR-342-3p was identified as a potential down-regulated biomarker of children aged 5–10 years with endothelial dysfunction [128]. Our study demonstrated decreased expression of miR-342-3p in a proportion of children with abnormal clinical findings that were previously exposed to early preeclampsia, which required the termination of gestation before 34 weeks (26.92%). Therefore, we suggest that this group of children is a highly risky group, which would benefit from implementation of early primary prevention strategies and long-term follow-up.

## 4. Materials and Methods

### 4.1. Participants

The study included prospectively collected cohort of Caucasian children born within 2007–2014 descending from pregnancies with GH (*n* = 54), PE (*n* = 133), FGR (*n* = 34), and children after normal course of gestation (*n* = 88) that were chosen on the basis of equal age. An in-person visit was conducted 3–11 years after the pregnancy ended. Of the 133 PE pregnancies, 27 were diagnosed with mild PE and 106 had symptoms of severe PE. In 49 PE pregnancies gestation was terminated before 34 weeks (early PE) and 84 children were delivered after 34 weeks (late PE). PE occurred mainly in normotensive patients (128 cases), or exceptionally was superimposed on prior hypertension (5 cases). Thirteen FGR fetuses required delivery <32 weeks (early FGR) and 21 cases were delivered >32 weeks (late FGR). Oligohydramnios and/or anhydramnios were present in 20 FGR fetuses and 19 PE cases.

Arterial Doppler examination showed an abnormal pulsatility index (PI) in the umbilical artery (PI > 95th percentile) in 9 PE and 20 FGR cases and in the middle cerebral artery (PI < 5th percentile) in 7 PE and 8 FGR cases. The cerebro-placental ratio (CPR) was 5th percentile in 15 PE and 21 FGR cases. The umbilical artery Doppler showed absent and/or zero diastolic flow in 2 PE and 4 FGR cases. The mean PI in the uterine artery >95th percentile was identified in 10 PE and 7 FGR pregnancies with the presence of unilateral or bilateral diastolic notch in 12 PE and 6 FGR cases. Ductus venosus examination revealed an absence of flow during atrial contraction (a wave) (deep a wave) in 1 FGR pregnancy. In addition, abnormal PI of ductus venosus (>1) was detected in 3 PE and/or FGR pregnancies.

The clinical characteristics of children descending from normal and complicated pregnancies are presented in Table 3.

Normal pregnancies were defined as those without medical, obstetrical, or surgical complications at the time of the study and who subsequently delivered full term, singleton healthy infants weighing >2500 g after 37 completed weeks of gestation.

Gestational hypertension usually develops after 20 weeks of gestation and is defined as high blood pressure (>140/90 mmHg) without the sign of proteinuria. On the other hand, preeclampsia is characterized as hypertension (blood pressure > 140/90 mmHg in two determinations 4 h apart) associated with proteinuria (>300 mg/24 h) that appears after the twentieth week of gestation [129].

Severe preeclampsia is defined by the presence of one or more of the following findings: 1) a systolic blood pressure over 160 mmHg or a diastolic blood pressure over 110 mmHg, 2) proteinuria (>5 g of protein in a 24-h sample), 3) very low urine output (<500 mL in 24 h), 4) signs of pulmonary oedema or cyanosis, 5) impairment of liver function, 6) signs of severe headache, visual disturbances, 7) pain in the epigastric area or right upper quadrant, 8) thrombocytopenia, and 9) the presence of severe FGR [129].

FGR fetuses are defined as those with the estimated fetal weight (EFW) < 3rd percentile or <10th percentile for the evaluated gestational age after the adjustments for the appropriate population standards of the Czech Republic (the Hadlock formula, Astraia Software GmbH). Early onset FGR was diagnosed when the EFW was less than the third percentile or absent and/or zero diastolic flow was present in the umbilical artery. In addition, early onset FGR was classified when fetal weight below the threshold of the 10th percentile was associated with an abnormal pulsatility index in the umbilical artery (>95th percentile) or an abnormal pulsatility index in the uterine artery (>95th percentile). Late onset FGR was determined by only one parameter (EWF below the third percentile) or by the combination of 2 parameters: EFW below the tenth percentile and the cerebro–placental ratio (CPR) below the fifth percentile. CPR is expressed as a ratio between the middle cerebral artery and the umbilical artery pulsatility indexes [130,131,132].

The presence of absent and/or zero end-diastolic flow (AEDF) in the umbilical artery in mid to late pregnancy usually occurs as a result of placental insufficiency. Increased resistance (the mean PI > 95th percentile) in the uterine artery with or without the presence of unilateral or bilateral diastolic notch identifies pregnancies with a risk of placental failure. Centralization of the fetal circulation manifests itself in redistribution of the circulation in the brain, liver and heart at the expense of the flow reduction in the periphery and represents a protective reaction of the fetus against hypoxia [133,134]. Absence or reversal of flow during atrial contraction (a wave) (deep a wave in the ductus venosus) indicates failure of fetal circulatory compensation to supply well oxygenated blood to vital organ. The pulsatility index of DV more than 1 between the second trimester and term indicates of DV dilatation and poor outcome in severe fetal growth retardation.

Patients demonstrating other pregnancy-related complications such as premature rupture of membranes, in utero infections, fetal anomalies or chromosomal abnormalities, and fetal demise in utero or stillbirth were not involved in the study.

Written informed consent was provided for all participants included in the study. The study was approved by the Ethics Committee of the Institute for the Care of the Mother and Child, Prague, Czech Republic (grant no. AZV 16-27761A, Long-term monitoring of complex cardiovascular profile in the mother, fetus and offspring descending from pregnancy-related complications, date of approval: 28 May 2015) and by the Ethics Committee of the Third Faculty of Medicine, Prague, Czech Republic (grant no. AZV 16-27761A, Long-term monitoring of complex cardiovascular profile in the mother, fetus and offspring descending from pregnancy-related complications, date of approval: 27 March 2014).

### 4.2. BP Measurements

Standardized BP measurements were performed. BP was measured three times in the right arm after a 5-min rest period during which the participant seated using an automated device (OMRON M6W, Omron Healthcare Co., Kyoto, Japan) and cuff for arm circumference 17–22 cm (OMRON CS). The average of the last 2 systolic and diastolic BP was used for the data analyses.

Normal BP was characterized as systolic blood pressure (SBP) and diastolic blood pressure (DBP) that were below the 90th percentile for gender, age, and height. Hypertension was diagnosed when average SBP or DBP reached on at least 3 separate occasions ≥95th percentile for gender, age, and height. Average SBP or DBP levels that were within the range of ≥90th percentile and <95th percentile were designated as prehypertension [135].

### 4.3. BMI Assessment

Body weight was measured to the nearest 0.05–0.1 kg using an electronic scale and height was measured to the nearest 0.1 cm using a built-in stadiometer (calibrated balance scales, RADWAG WPT 100/200 OW, RADWAG, Czech Republic). The BMI Percentile Calculator was used to calculate BMI in children and teens (https://www.cdc.gov/healthyweight/assessing/bmi/childrens_bmi/about_childrens_bmi.html).

This calculator provides age- and sex-specific BMI. Normal or healthy weight children had BMI within the range of the 5th percentile and the 85th percentile. Children with BMI within the 85th percentile and the 95th percentile were in the overweight category. Children with BMI equal to or greater than the 95th percentile were in the obese category.

### 4.4. Echocardiography Measurements

Examinations were performed using Philips HD15 ultrasound machine (Philips Ultrasound, Bothell, WA, USA) with sector array transducer (3–8 MHz) incorporating color flow, pulse wave, and continuous wave Doppler measurements with adaptive technology. Children were calm and in supine position during ultrasound examination. A complete two-dimensional echocardiography was performed by a single investigator experienced with pediatric echocardiography. The transducer beam was kept as close as possible to the Doppler beam at <20% degrees to calculate valve regurgitation. No angle correction of Doppler signal was applied. Children with abnormal findings were referred to pediatric cardiologist.

### 4.5. Processing of Samples

Homogenized cell lysates were prepared immediately after collection of whole peripheral blood samples (EDTA tubes, 200 µL) using QIAamp RNA Blood Mini Kit (Qiagen, Hilden, Germany, no: 52304).

Total RNA was extracted from homogenized cell lysates stored at −80 °C using a mirVana microRNA Isolation kit (Ambion, Austin, USA, no: AM1560) and followed by an enrichment procedure for small RNAs. To minimize DNA contamination, the eluted RNA was treated for 30 min at 37 °C with 5 µL of DNase I (Thermo Fisher Scientific, CA, USA, no: EN0521). A RNA fraction highly enriched in short RNAs (<200 nt) was obtained. The concentration and quality of RNA was assessed using a NanoDrop ND-1000 spectrophotometer (NanoDrop Technologies, Wilmington, NC, USA). If the A(260/280) absorbance ratio of isolated RNA was 1.8–2.0 and the A(260/230) absorbance ratio was greater than 1.6, the RNA fraction was pure and used for the consecutive analysis.

### 4.6. Reverse Transcriptase Reaction

Individual microRNAs were reverse transcribed into complementary DNA (cDNA) in a total reaction volume of 10 µL using microRNA-specific stem-loop RT primers, components of TaqMan MicroRNA Assays (Table 4), and TaqMan MicroRNA Reverse Transcription Kit (Applied Biosystems, Branchburg, NJ, USA, no: 4366597). Reverse transcriptase reactions were performed with the following thermal cycling parameters: 30 min at 16 °C, 30 min at 42 °C, 5 min at 85 °C, and then held at 4 °C using a 7500 Real-Time PCR system (Applied Biosystems, Branchburg, NJ, USA).

### 4.7. Relative Quantification of microRNAs by Real-Time PCR

3 µL of cDNA were mixed with specific TaqMan MGB probes and primers (TaqMan MicroRNA Assay, Applied Biosystems, Branchburg, NJ, USA), and the ingredients of the TaqMan Universal PCR Master Mix (Applied Biosystems, Branchburg, NJ, USA, no: 4318157). A total reaction volume was 15 µL. TaqMan PCR conditions were set up as described in the TaqMan guidelines for a 7500 Real-Time PCR system. All PCRs were performed in duplicates with the involvement of multiple negative controls such as NTC (water instead of cDNA sample), NAC (non-transcribed RNA samples), and genomic DNA (isolated from equal biological samples), which did not generate any signal during PCR reactions. The samples were considered positive if the amplification signal occurred at *Ct* < 40 (before the 40th threshold cycle).

The expression of particular microRNA was determined using the comparative Ct method [136] relative to normalization factor (geometric mean of two selected endogenous controls) [137]. Two non-coding small nucleolar RNAs (RNU58A and RNU38B) were optimal for qPCR data normalization in this setting. They demonstrated equal expression between children descending from normal and complicated pregnancies. RNU58A and RNU38B also served as positive controls for successful extraction of RNA from all samples and were used as internal controls for variations during the preparation of RNA, cDNA synthesis, and real-time PCR.

A reference sample, RNA fraction highly enriched for small RNAs isolated from the fetal part of one randomly selected placenta derived from gestation with normal course, was used throughout the study for relative quantification.

### 4.8. Statistical Analysis

Data normality was assessed using the Shapiro–Wilk test [138]. Since our experimental data did not follow a normal distribution, microRNA levels were compared between groups using the Kruskal–Wallis one-way analysis of variance with post-hoc test for the comparison among multiple groups. The significance level was established at a *p*-value of *p* < 0.05.

Receivers operating characteristic (ROC) curves were constructed to calculate the area under the curve (AUC) and the best cut-off point for particular microRNA was used in order to calculate the respective sensitivity at 90.0% specificity (MedCalc Software bvba, Ostend, Belgium). For every possible threshold or cut-off value, the MedCalc^®^ v16.8.4 program reports the sensitivity, specificity, likelihood ratio positive (LR+), likelihood ratio negative (LR−).

To select the optimal combinations of microRNA biomarkers logistic regression was used (MedCalc^®^ v16.8.4 program, MedCalc Software bvba, Ostend, Belgium). The logistic regression procedure allows to analyze the relationship between one dichotomous dependent variable and one or more independent variables. Another method to evaluate the logistic regression model makes use of ROC curve analysis. In this analysis, the power of the model’s predicted values to discriminate between positive and negative cases is quantified by the area under the ROC curve (AUC). To perform a full ROC curve analysis the predicted probabilities are first saved and next used as a new variable in ROC curve analysis. The dependent variable used in logistic regression then acts as the classification variable in the ROC curve analysis dialog box.

Correlation between variables was calculated using the Spearman’s rank correlation coefficient (ρ). Spearman’s rank correlation coefficient, a nonparametric measure of rank correlation, assesses how well the relationship between two variables can be described using a monotonic function.

If the correlation coefficient value ranges within <0.5; 1.0>, there is a strong positive correlation. The significance level was established at a *p*-value of *p* < 0.05.

Box plots encompassing the median (dark horizontal line) of log-normalized gene expression values for particular microRNAs were generated using Statistica software (version 9.0; StatSoft, Inc., Tulsa, OK, USA). The upper and lower limits of the boxes represent the 75th and 25th percentiles, respectively. The upper and lower whiskers indicate the maximum and minimum values that are no more than 1.5 times the span of the interquartile range (range of the values between the 25th and the 75th percentiles). Outliers are marked by circles and extremes by asterisks.

## 5. Conclusions

In conclusion, epigenetic changes characteristic for cardiovascular/cerebrovascular diseases are also present in children descending from complicated pregnancies. Previous occurrence of GH, PE, or FGR may predispose to later development of cardiovascular/cerebrovascular diseases in offspring. Consecutive large scale studies including the children with a single clinical entity are needed to verify the findings resulting from this particular pilot study.

## 6. Patents

National Patent Application-Industrial Property Office, Czech Republic (PV 2018-595).

## Figures and Tables

**Table 1:** Aberrant expression profile of microRNAs in children descending from pregnancy-related complications.

MicroRNA Expression in Children Descending from Pregnancy-Related Complications			
miRBase ID	Gestational Hypertension (GH)	Preeclampsia (PE)	Fetal Growth Restriction (FGR)
hsa-miR-1-3p	↑ children with both normal and abnormal clinical findings	↑ late PE, only children with abnormal clinical findings	
hsa-miR-17-5p	↑ children with both normal and abnormal clinical findings		↑ only children with abnormal clinical findings
hsa-miR-20a-5p	↑ only children with abnormal clinical findings	↑ late PE, only children with abnormal clinical findings	
hsa-miR-20b-5p		↑ mild PE, only children with normal clinical findings	
hsa-miR-21-5p	↑ only children with normal clinical findings		
hsa-miR-23a-3p	↑ only children with normal clinical findings		
hsa-miR-26a-5p	↑ only children with normal clinical findings		
hsa-miR-29a-3p	↑ children with both normal and abnormal clinical findings		
hsa-miR-103a-3p		↑ severe PE, late PE, only children with abnormal clinical findings	
hsa-miR-125b-5p	↑ only children with normal clinical findings		
hsa-miR-126-3p	↑ children with both normal and abnormal clinical findings		↑ only children with abnormal clinical findings
hsa-miR-133a-3p	↑ children with both normal and abnormal clinical findings	↑ PE, severe PE, late PE children with both normal and abnormal clinical findings early PE, only children with normal clinical findings	↑ only children with abnormal clinical findings
hsa-miR-146a-5p	↑ children with both normal and abnormal clinical findings		
hsa-miR-181a-5p	↑ children with both normal and abnormal clinical findings		
hsa-miR-195-5p	↑ only children with normal clinical findings		
hsa-miR-210-3p		↑ children descending from PE and/or FGR complicated pregnancies with increased PI in the ductus venosus during gestation	
hsa-miR-342-3p		↓ early PE, only children with abnormal clinical findings	

↑ increased expression of microRNA, ↓ decreased expression of microRNA.

**Table 2:** The role of differentially expressed microRNAs in children descending from gestational hypertension, preeclampsia and/or fetal growth restriction complicated pregnancies in the pathogenesis of cardiovascular/cerebrovascular diseases.

miRBase ID	Gene Location on Chromosome	Expression	Role in the Pathogenesis of Cardiovascular/Cerebrovascular Diseases	Potential Therapeutic Target in Treatment of Cardiovascular Diseases
hsa-miR-1-3p	20q13.3 18q11.2 [46]	Cardiac and skeletal muscles, myocardium	Acute myocardial infarction, heart ischemia, post-myocardial infarction complications [47]	+ [48,49,50]
hsa-miR-17-5p	13q31.3 [51,52]	Endothelial cells, vascular smooth muscle cells [53]	Cardiac development [54], ischemia/reperfusion-induced cardiac injury [55], kidney ischemia-reperfusion injury [57], diffuse myocardial fibrosis in hypertrophic cardiomyopathy [58], acute ischemic stroke [59], coronary artery disease [60]	+ [55,56]
hsa-miR-20a-5p	13q31.3 [61]	Pulmonary arteries [62]	Pulmonary hypertension [62], gestational diabetes mellitus [63]	+ [62]
hsa-miR-20b-5p	Xq26.2 [61]		Hypertension-induced heart failure [64], small for gestational age foetuses [65]	
hsa-miR-21-5p	17q23.2 [66]	Cardiomyocytes	Homeostasis of the cardiovascular system [67], cardiac fibrosis and heart failure [68,70]	+ [68,69]
hsa-miR-23a-3p	19p13.12	Cardiomyocytes	Heart failure [71], coronary artery disease [72], cerebral ischemia-reperfusion [73]	
hsa-miR-26a-5p	3p22.2 12q14.1 [74]	Cardiac fibroblasts [75]	Heart failure, cardiac hypertrophy [75]	
hsa-miR-29a-3p	7q32.3	Heart	Ischemia/reperfusion-induced cardiac injury [76], cardiac cachexia, heart failure [77], atrial fibrillation [78], diffuse myocardial fibrosis in hypertrophic cardiomyopathy [58], gestational diabetes mellitus [63], T2DM [23,80]	+ [76]
hsa-miR-103a-3p	5q34 20p13 [81]	Heart Pulmonary arterial smooth muscle cells	Hypertension [82], hypoxia-induced pulmonary hypertension [84], myocardial ischemia/reperfusion injury, acute myocardial infarction [82], obesity, regulation of insulin sensitivity [85]	+ [83]
hsa-miR-125b-5p	11q24.1 21q21.1 [86]	Endothelial cells [89], cardiomyocytes [90]	Acute ischemic stroke [87], acute myocardial infarction [88,90]	
hsa-miR-126-3p	9q34.3 [91]	Endothelial cells [21], vascular smooth muscle cells [95]	Acute myocardial infarction [93], T2DM [94]	+ [21,95]
hsa-miR-133a-3p	18q11.2 20q13.33 [96]	Heart	Heart failure [98], myocardial fibrosis in hypertrophic cardiomyopathy [58,97], arrhythmogenesis in the hypertrophic and failing hearts [99,100], coronary artery calcification [102]	+ [99,100]
hsa-miR-146a-5p	5q33.3 [103,104]	Myocardium, brain	Angiogenesis [105], hypoxia, ischemia/reperfusion-induced cardiac injury [107], coronary atherosclerosis, coronary heart disease in patients with subclinical hypothyroidism [108], acute ischemic stroke, acute cerebral ischemia [106]	+ [107]
hsa-miR-181a-5p	1q32.1 9q33.3 [109]	Monocytes, adipocytes, hepatocytes	Atherosclerosis [109], T1DM [113], T2DM [109,111], obesity [109,110,111], metabolic syndrome, coronary artery disease [110], insulin resistance [111], non-alcoholic fatty liver disease [112], ischaemic stroke, transient ischaemic attack, acute myocardial infarction [114,115]	
hsa-miR-195-5p	17p13.1 [116]	Aorta, abdominal aorta	Cardiac hypertrophy, heart failure [29,118], abdominal aortic aneurysms [119], aortic stenosis [120]	+ [29,118]
hsa-miR-210-3p	11p15.5	Endothelial cells, cardiomyocytes [125], skeletal muscle [124]	Hypoxia [39], atherosclerotic plaque formation [25,123,126], heart failure [127], cerebral ischemia [128]	
hsa-miR-342-3p	14q32.2	Endothelial cells	Obesity [129], T1DM [130,133], T2DM [130,131,132], GDM [130], endothelial dysfunction [134]	

+ Potential Therapeutic Target in Treatment of Cardiovascular Diseases.

**Table 3:** Characteristics of cases and controls.

	Normal Pregnancies with Normal Clinical Findings (*n* = 50)	Normal Pregnancies with Abnormal Clinical Findings (*n* = 38)	PE (*n* = 133)	FGR (*n* = 34)	GH (*n* = 54)	*p*-Value ^1^	*p*-Value ^2^	*p*-Value ^3^	*p*-Value ^4^
**At follow-up**									
Age (years)	5 (3–11)	5 (3–11)	5 (3–11)	4 (3–10)	4.5 (3–10)	1.000	1.000	1.000	1.000
Height (cm)	115 (98–144.5)	118.5 (100–153)	114 (97–155)	106.5 (93–152)	111.5 (96–159.5)	1.000	1.000	**0.020**	1.000
Weight (kg)	20.35 (14–37)	22.3 (14.7–40.8)	19.4 (11.85–54.9)	16.25 (12–37)	19.6 (14–47.5)	1.000	1.000	**0.002**	1.000
BMI (kg/m^2^)	15.43 (13.22–18.09)	15.87 (13.3–20)	14.91 (12.34–22.81)	14.18 (12.7–19.24)	15.35 (13.42–19.7)	1.000	1.000	**0.004**	1.000
Systolic BP (mmHg)	98 (84–115)	104 (89–123)	99 (84–132)	97 (82–123)	99 (80–129)	**0.001**	1.000	1.000	0.487
Diastolic BP (mmHg)	60 (38–68)	64.4 (43–81)	61 (41–88)	60 (42–75)	61.5 (49–83)	**0.028**	0.545	1.000	1.000
Heart rate (n/min)	90 (67–110)	90.5 (51–120)	92 (64–117)	96 (62–112)	94.5 (65–129)	1.000	1.000	1.000	1.000
**During gestation**									
Maternal age at delivery (years)	32.5 (26–40)	32 (25–43)	32 (21–44)	32 (22–41)	32 (27–51)	1.000	1.000	1.000	1.000
GA at delivery (weeks)	39.86 (37.71–41.57)	39.93 (37.86–41.86)	35.79 (26–41.72)	35.64 (28–41)	38.63 (33.43–41.28)	1.000	**<0.001**	**<0.001**	**0.002**
**Mode of delivery**						**0.429**	**<0.001**	**<0.001**	**<0.001**
Vaginal	46 (92.00 %)	33 (68.84%)	8 (14.3 %)	7 (20.59%)	24 (44.44%)				
CS	4 (8.00 %)	5 (13.16%)	48 (85.7 %)	27 (79.41%)	30 (55.56%)				
Fetal birth weight (g)	3425 (2730–4220)	3295 (2530–4450)	2370 (660–4490)	1870 (650-3010)	3140 (1040-4310)	1.000	**<0.001**	**<0.001**	0.113
**Fetal sex**						0.217	0.055	0.470	0.414
Boy	29 (58.00%)	17 (44.74%)	56 (42.11%)	17 (50.00%)	27 (50.00%)				
Girl	21 (42.00 %)	21 (55.26%)	77 (57.89%)	17 (50.00%)	27 (50.00%)				
Primiparity						0.140	**0.001**	**<0.001**	0.362
Yes	29 (58.00%)	16 (42.11%)	108 (81.20%)	33 (97.06%)	36 (66.67 %)				
No	21 (42.00%)	22 (57.89%)	25 (18.80 %)	1 (2.94 %)	18 (33.33 %)				
**Birth order of index pregnancy**						0.158	0.168	**0.009**	0.602
1st	25 (50.00%)	12 (31.58%)	86 (64.66%)	28 (82.35)	28 (51.85%)				
2nd	18 (36.00%)	14 (36.84%)	27 (20.30%)	2 (5.88%)	14 (25.93%)				
3rd	5 (10.00%)	10 (26.32%)	13 (9.77 %)	2 (5.88%)	9 (16.66 %)				
4th+	2 (4.00 %)	2 (5.26%)	7 (5.26 %)	2 (5.88%)	3 (5.56 %)				
**Infertility treatment**						0.726	**0.001**	**0.007**	0.117
Yes	2 (4.00%)	1 (2.63%)	34 (25.56%)	8 (23.53 %)	7 (12.96%)				
No	48 (96.00%)	37 (97.37%)	99 (74.44%)	26 (76.47 %)	47 (87.04%)				

Data are presented as median (range) for continuous variables and as number (percent) for categorical variables. Statistically significant results are marked in bold. Continuous variables were compared using Kruskal-Wallis test. *p*-value ^1^: the comparison among normal pregnancies with normal and abnormal postnatal clinical findings; *p*-value ^2, 3, 4^: the comparison among normal pregnancies with normal postnatal clinical findings and preeclampsia, fetal growth restriction or gestational hypertension, respectively. Categorical variables were compared using a chi-square test.; GA, gestational age; BP, blood pressure; CS, Caesarean section.

**Table 4:** Characteristics of microRNAs involved in the study.

Assay Name	miRBase ID	NCBI Location Chromosome	microRNA Sequence
hsa-miR-1	hsa-miR-1-3p	Chr20: 61151513-61151583 [+]	5′-UGGAAUGUAAAGAAGUAUGUAU-3′
hsa-miR-16	hsa-miR-16-5p	Chr13: 50623109-50623197 [−]	5′-UAGCAGCACGUAAAUAUUGGCG- 3′
hsa-miR-17	hsa-miR-17-5p	Chr13: 92002859-92002942 [+]	5′-CAAAGUGCUUACAGUGCAGGUAG-3′
hsa-miR-20a	hsa-miR-20a-5p	Chr13: 92003319-92003389 [+]	5′-UAAAGUGCUUAUAGUGCAGGUAG-3′
hsa-miR-20b	hsa-miR-20b-5p	ChrX: 133303839-133303907 [−]	5′-CAAAGUGCUCAUAGUGCAGGUAG-3′
hsa-miR-21	hsa-miR-21-5p	Chr17: 57918627-57918698 [+]	5′-UAGCUUAUCAGACUGAUGUUGA-3′
hsa-miR-23a	hsa-miR-23a-3p	Chr19: 13947401-13947473 [−]	5′-AUCACAUUGCCAGGGAUUUCC-3′
hsa-miR-24	hsa-miR-24-3p	Chr19: 13947101-13947173 [−]	5′-UGGCUCAGUUCAGCAGGAACAG-3′
hsa-miR-26a	hsa-miR-26a-5p	Chr3: 38010895-38010971 [+]	5′-UUCAAGUAAUCCAGGAUAGGCU-3′
hsa-miR-29a	hsa-miR-29a-3p	Chr7: 130561506-130561569 [−]	5′-UAGCACCAUCUGAAAUCGGUUA-3′
hsa-miR-92a	hsa-miR-92a-3p	Chr13: 92003568-92003645 [+]	5′-UAUUGCACUUGUCCCGGCCUGU-3′
hsa-miR-100	hsa-miR-100-5p	Chr11: 122022937-122023016 [−]	5′-AACCCGUAGAUCCGAACUUGUG-3′
hsa-miR-103	hsa-miR-103a-3p	Chr20: 3898141-3898218 [+]	5′-AGCAGCAUUGUACAGGGCUAUGA-3′
hsa-miR-125b	hsa-miR-125b-5p	Chr21: 17962557-17962645 [+]	5′-UCCCUGAGACCCUAACUUGUGA-3′
hsa-miR-126	hsa-miR-126-3p	Chr9: 139565054-139565138 [+]	5′-UCGUACCGUGAGUAAUAAUGCG-3′
hsa-miR-130b	hsa-miR-130b-3p	Chr22: 22007593-22007674 [+]	5′-CAGUGCAAUGAUGAAAGGGCAU-3′
hsa-miR-133a	hsa-miR-133a-3p	Chr20: 61162119-61162220 [+]	5′-UUUGGUCCCCUUCAACCAGCUG-3′
hsa-miR-143	hsa-miR-143-3p	Chr5: 148808481-148808586 [+]	5′-UGAGAUGAAGCACUGUAGCUC-3′
hsa-miR-145	hsa-miR-145-5p	Chr5: 148810209-148810296 [+]	5′-GUCCAGUUUUCCCAGGAAUCCCU-3′
hsa-miR-146a	hsa-miR-146a-5p	Chr5: 159912359-159912457 [+]	5′-UGAGAACUGAAUUCCAUGGGUU-3′
hsa-miR-155	hsa-miR-155-5p	Chr21: 26946292-26946356 [+]	5′-UUAAUGCUAAUCGUGAUAGGGGU-3′
hsa-miR-181a	hsa-miR-181a-5p	Chr9: 127454721-127454830 [+]	5′-AACAUUCAACGCUGUCGGUGAGU-3′
hsa-miR-195	hsa-miR-195-5p	Chr17: 6920934-6921020 [−]	5′-UAGCAGCACAGAAAUAUUGGC-3′
hsa-miR-199a	hsa-miR-199a-5p	Chr19: 10928102-10928172 [−]	5′-CCCAGUGUUCAGACUACCUGUUC-3′
hsa-miR-210	hsa-miR-210-3p	Chr11: 568089-568198 [−]	5′-CUGUGCGUGUGACAGCGGCUGA-3′
hsa-miR-221	hsa-miR-221-3p	ChrX: 45605585-45605694 [−]	5′-AGCUACAUUGUCUGCUGGGUUUC-3′
hsa-miR-342-3p	hsa-miR-342-3p	Chr14: 100575992-100576090 [+]	5′-UCUCACACAGAAAUCGCACCCGU-3′
mmu-miR-499	hsa-miR-499a-5p	Chr20: 33578179-33578300 [+]	5′-UUAAGACUUGCAGUGAUGUUU-3′
hsa-miR-574-3p	hsa-miR-574-3p	Chr4: 38869653-38869748 [+]	5′-CACGCUCAUGCACACACCCACA-3′

[+] A single strand of DNA sense (or positive (+)) if an RNA version of the same sequence is translated or translatable into protein. [−] Its complementary strand is called antisense (or negative (−) sense).

## Supplementary Materials

Supplementary materials can be found at http://www.mdpi.com/1422-0067/20/3/654/s1.

## Abbreviations

PE	Preeclampsia
FGR	Fetal growth restriction
GH	Gestational hypertension
DV	Ductus venosus
CPR	Cerebro-placental ratio
PI	Pulsatility index
BMI	Body mass index
