# Supplementary material for: Postnatal Expression Profile of microRNAs Associated with Cardiovascular and Cerebrovascular Diseases in Children at the Age of 3 to 11 Years in Relation to Previous Occurrence of Pregnancy-Related Complications
Source: Int J Mol Sci. 2019 Feb 2;20(3):654. doi: 10.3390/ijms20030654 (PMC6387366; doi:10.3390/ijms20030654)
Supplement: Supplementary file 1 [file ijms-20-00654-s001.pdf]

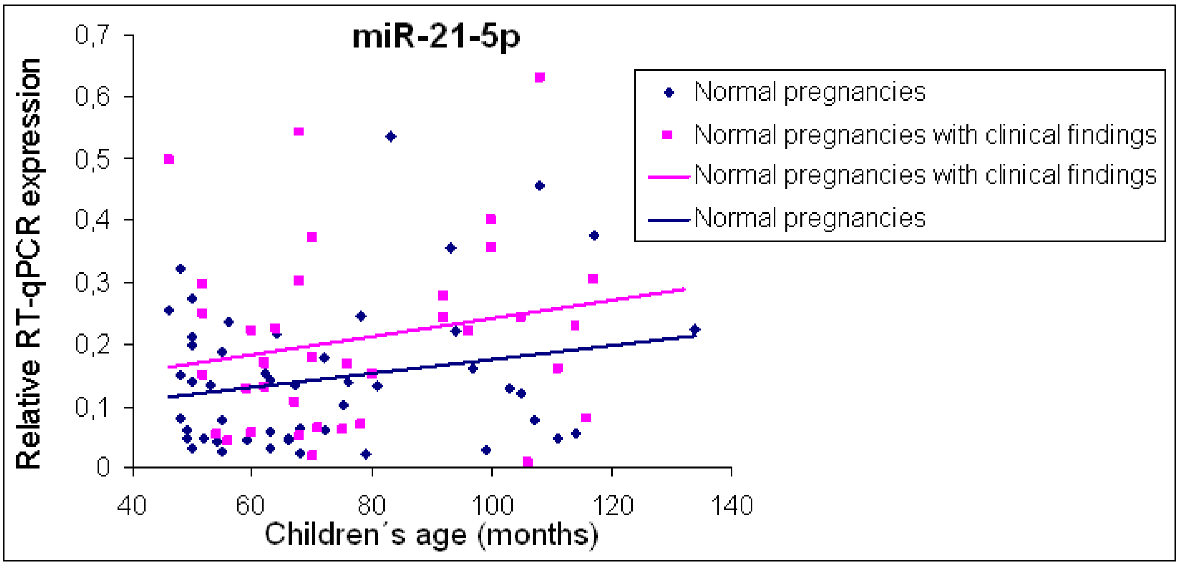

Figure S1. The effect of children age on miR-21-5p expression in children descending from uncomplicated pregnancies. We are showing the examples of behaviour of microRNAs in relation to children age in particular situations. For instance, in case of miR-21-5p, the gene expression was constantly increasing with an advancing age of children similarly in both groups, and therefore a quite constant difference in gene expression of miR-21-5p within control groups with normal and abnormal clinical findings could be observed.

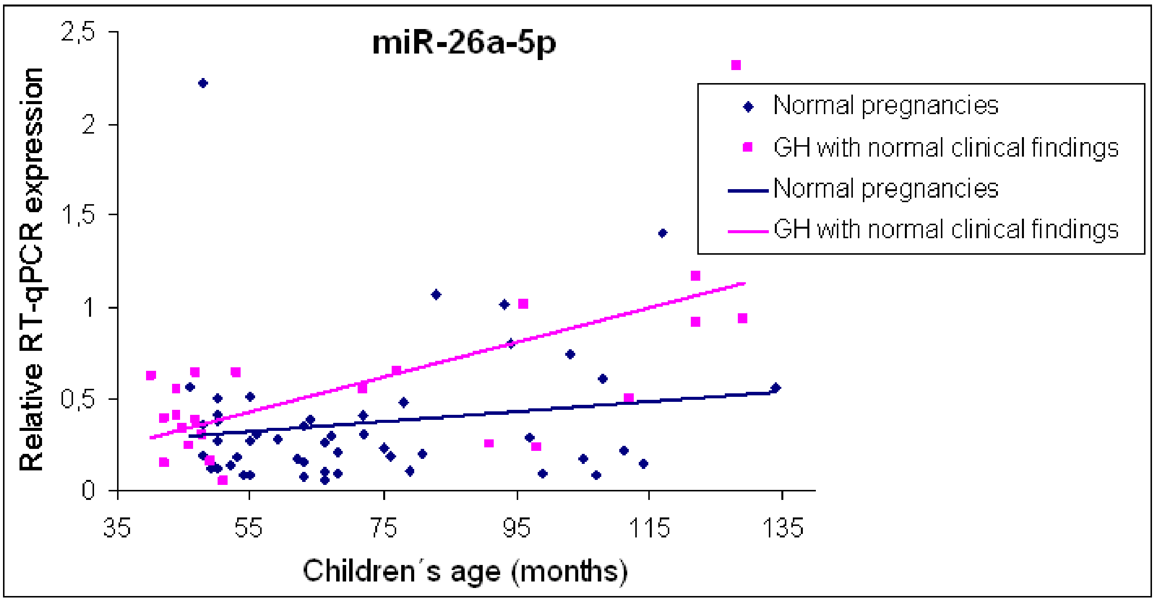

Figure S2. The effect of children age on miR-26a-5p expression in children descending from normal and GH complicated pregnancies. We are showing the examples of behaviour of microRNAs in relation to children age in particular situations. For instance, in case of miR-26a-5p we could observe an increase of gene expression with advancing age of children in both groups (children descending from normal and GH complicated pregnancies) and an increasing difference in microRNA expression between controls and a group of GH complicated pregnancies with an advancing age of children.

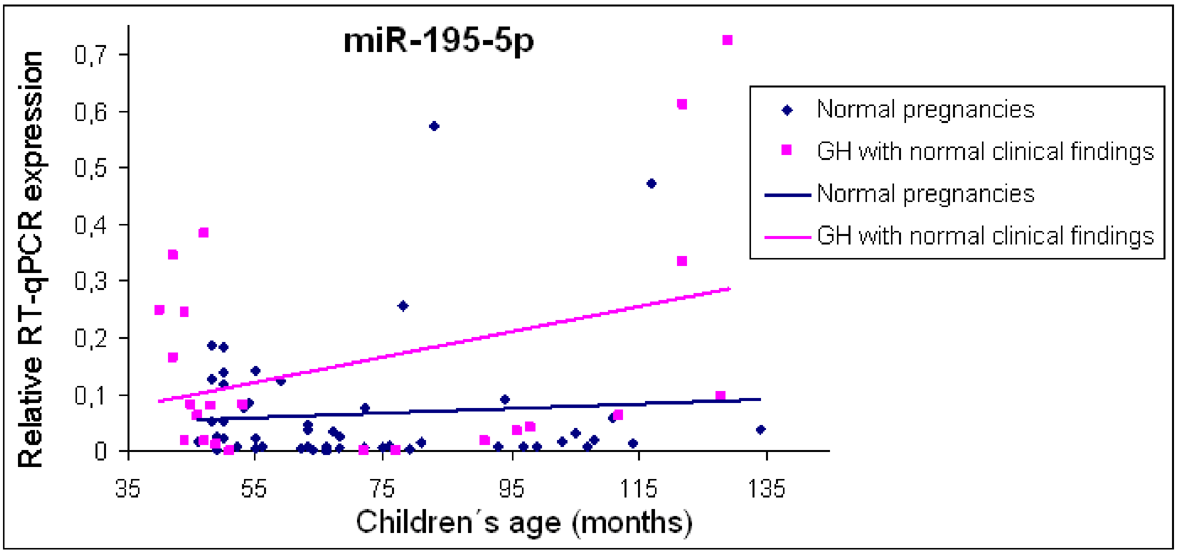

Figure S3. The effect of children age on miR-195-5p expression in children descending from normal and GH complicated pregnancies. We are showing the examples of behaviour of microRNAs in relation to children age in particular situations. For instance, in case of miR-195-5p we could observe an increase of gene expression with advancing age of children in both groups (children descending from normal and GH complicated pregnancies) and an increasing difference in microRNA expression between controls and a group of GH complicated pregnancies with an advancing age of children.

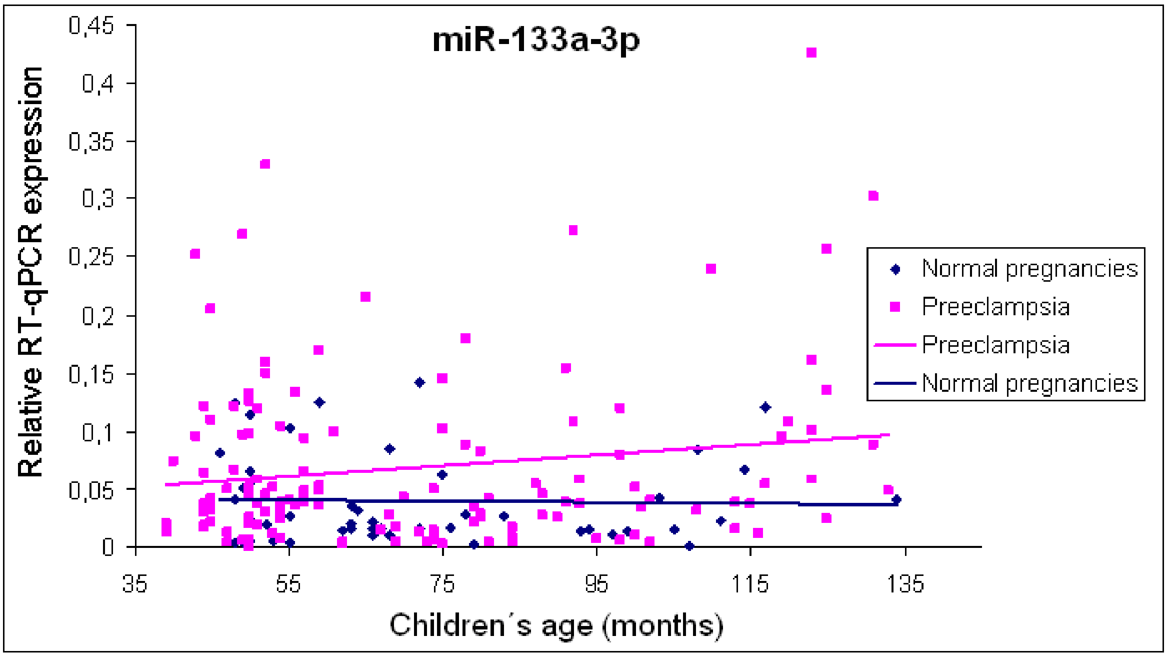

Figure S4. The effect of children age on miR-133a-3p expression in children descending from normal and PE complicated pregnancies. We are showing the examples of behaviour of microRNAs in relation to children age in particular situations. For instance, in case of miR-133a-3p we could observe a sign of decrease of expression with advancing age of children in offspring descending from normal pregnancies, and an increase of expression with advancing age of children in offspring descending from PE affected pregnancies. As a consequence an increasing difference in gene expression of miR-133a-3p between the controls and a group of PE complicated pregnancies could be observed.
